# Generating interacting protein sequences using domain-to-domain translation

**DOI:** 10.1093/bioinformatics/btad401

**Published:** 2023-07-03

**Authors:** Barthelemy Meynard-Piganeau, Caterina Fabbri, Martin Weigt, Andrea Pagnani, Christoph Feinauer

**Affiliations:** Computational and Quantitative Biology, LCQB UMR 7238, Institut de Biologie Paris Seine, CNRS, Sorbonne Université, Paris 75005, France; Department of Computing Sciences, Bocconi University, Milan 20100, Italy; Department of Computing Sciences, Bocconi University, Milan 20100, Italy; Computational and Quantitative Biology, LCQB UMR 7238, Institut de Biologie Paris Seine, CNRS, Sorbonne Université, Paris 75005, France; Politecnico di Torino, Duca degli Abruzzi, 24, Turin 10129, Italy; Italian Institute for Genomic Medicine, Strada Provinciale, 142, Candiolo 10060, Italy; Department of Computing Sciences, Bocconi University, Milan 20100, Italy

## Abstract

**Motivation:**

Being able to artificially design novel proteins of desired function is pivotal in many biological and biomedical applications. Generative statistical modeling has recently emerged as a new paradigm for designing amino acid sequences, including in particular models and embedding methods borrowed from natural language processing (NLP). However, most approaches target single proteins or protein domains, and do not take into account any functional specificity or interaction with the context. To extend beyond current computational strategies, we develop a method for generating protein domain sequences intended to interact with another protein domain. Using data from natural multidomain proteins, we cast the problem as a translation problem from a given interactor domain to the new domain to be generated, i.e. we generate artificial partner sequences conditional on an input sequence. We also show in an example that the same procedure can be applied to interactions between distinct proteins.

**Results:**

Evaluating our model’s quality using diverse metrics, in part related to distinct biological questions, we show that our method outperforms state-of-the-art shallow autoregressive strategies. We also explore the possibility of fine-tuning pretrained large language models for the same task and of using Alphafold 2 for assessing the quality of sampled sequences.

**Availability and implementation:**

Data and code on https://github.com/barthelemymp/Domain2DomainProteinTranslation.

## 1 Introduction

Generating novel protein sequences with desired properties is one of the key challenges of computational biology. It is likely that machine learning methods will play an important role in this task, being already used for the generation of new enzymes, biological sensors, and drug molecules (Wu *et al.* 2021). A promising approach is to leverage deep generative models, which use neural networks for learning probability distributions from known, naturally occurring protein sequences (Alley *et al.* 2019, Madani *et al.* 2020, Hawkins-Hooker *et al.* 2021, Shin *et al.* 2021, Repecka *et al.* 2021). Apart from other uses, like the prediction of mutational effects (Riesselman *et al.* 2018), these models can be used for protein design by selecting high-probability sequences (possibly under constraints) from the learned distribution.

Naturally occurring protein sequences are often comprised of several domains, and domains can be classified into different families (Alberts 2008). Models that work on the domain level usually use as training data a single multiple sequence alignment (MSA) (Durbin *et al.* 1998), containing sequences from the same domain family after aligning them, and make the assumption that each sequence is constrained by the same fitness landscape. This modeling paradigm neglects the dependence of the sequence constraints on the specific context corresponding to each organism, including other proteins interacting with the sequence or other domains on the same protein. Together with the fact that most of the crystallographic structures deposited in the PDB database (Burley *et al.* 2017) are resolved only at the single domain level (Zhou *et al.* 2022), this poses interesting questions about the limitations of current approaches, e.g. when predicting the relative orientation of multidomain proteins (Wu *et al.* 2021). Another field where this issue arises is immunology, where monoclonal antibody experiments are typically performed on mouse models and only later tested in humans. This is related to the so-called humanization problem, i.e. how to graft a promising variable receptor region (CDR) from a murine to a human context (Clavero-Álvarez *et al.* 2018). For protein design, this approach may be especially relevant. When redesigning a protein in order to increase its fitness, one usually only has to redesign a specific active domain inside the protein (Cheng *et al.* 2014, Reimer *et al.* 2019, Marchand *et al.* 2022). Being able to condition this process on the context (like, e.g. interacting domain inside the protein, or interacting domain of another protein) could potentially improve the precision of the design.

Known families of interacting domains can be organized in a paired MSA (pMSA), where the aligned interaction partners are concatenated (Muscat *et al.* 2020). Given the evolutionary pressure for maintaining functional interactions between proteins, amino acid substitutions at interaction surfaces are not independent between the interaction partners. The interacting sequence therefore can be used as additional information when generating a novel sequence. The current work addresses the task of generating domain sequences given an interacting domain sequence. Given that this task is similar to translation tasks in natural language processing (NLP), we explore the use of Transformers in this context. While there is some recent work using Transformers for translating between protein sequences (Wu *et al.* 2020) for specific applications, there is, to the best of our knowledge, no systematic exploration of this idea on the level of protein domain families on a diverse dataset. We explore different architectural choices, and regularization schemes and compare our results with a recently published shallow autoregressive method (Trinquier *et al.* 2021), which we use as a baseline. We also compare on a smaller scale to fine-tuned large protein language models, using Rita (Hesslow *et al.* 2022), and explore how structural predictions from Alphafold 2 correlate with our results.

**Figure 1.**
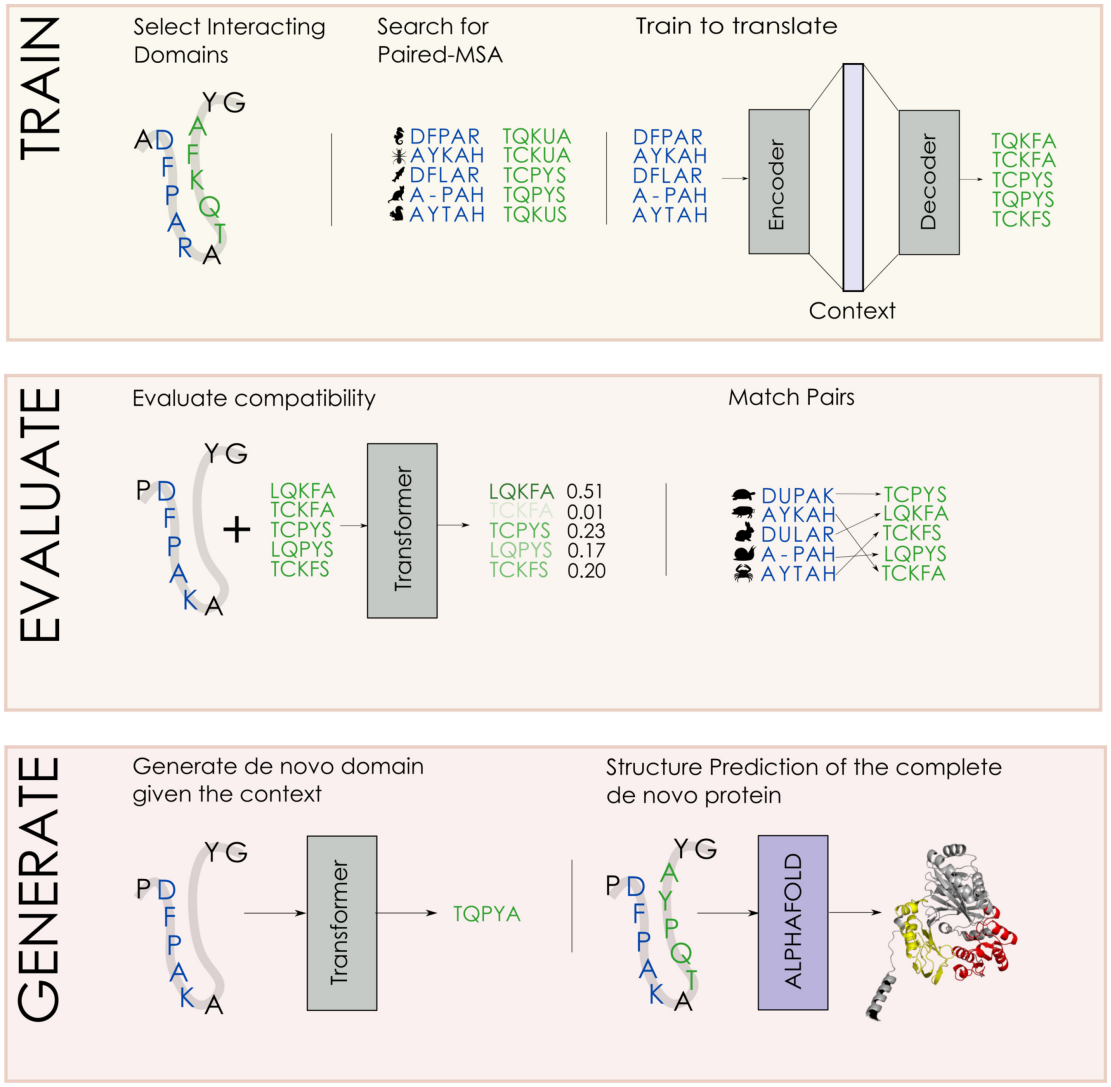
Summary of the work presented in this article. In the first box (Train), we extract interacting domains from known structures. We then build a pMSA based on homologous sequences of these domains and train the Transformer to translate between them. In the second box (Evaluate), we use the probabilities of the trained Transformers to match the source and target domains and assess the resulting accuracy. In the third box (Generate), we sample novel target domains and use them for replacing the original target domain. We then use Alphafold to predict the structure of the modified sequence and analyze the difference to the original structure.

The general idea of this work is summarized in Fig. 1. Consider a protein with at least two interacting domains, where interaction is defined as having a pair of amino acids at a distance of less than 8 angstrom. We then search a database of proteins for other sequences where these domains co-occur in the same protein and assemble the pMSA and use it for training the Transformer to translate from one domain to the other. The decoder being a causal language model, we can efficiently calculate the probability of a target sequence given the input sequence. This probability enables us to evaluate the compatibility of domains, which can be used for matching a domain to an interacting partner among several possible partners. The model is generative in that it can be used for generating a novel target sequence given the input sequence. Given a context, we can generate a new “translation” or target sequence and evaluate the new *de novo* proteins. In this setting, we highlight that one model per pair of domains is trained. We intend this article to fit into the line of work of domain-specific models, like in Potts Models, Variational Autoencoders (VAEs), and Restricted Boltzmann Machines (RBMs) (Tubiana *et al.* 2019, Russ *et al.* 2020, Hawkins-Hooker *et al.* 2021). We intend to provide a method for the task of redesigning a specific domain/protein, e.g. to increase its specific fitness for a specific task. We also explore the possibility of training one large Transformer for all the pairs, without observing any clear transfer learning advantage cf. Supplementary Appendix Section B.4.

## 2 Related literature

Generative modeling for protein design has a wide range of applications and a considerable number of different models have been proposed in the literature, recently especially deep neural network models (Wu *et al.* 2021). These include autoregressive models based on convolutional architectures (Shin *et al.* 2021), generative adversarial networks (Repecka *et al.* 2021), variational autoencoders (Hawkins-Hooker *et al.* 2021), LSTM-based architectures (Alley *et al.* 2019), and self-attention-based architectures (Madani *et al.* 2020). The latter work allows for sequence generation conditioned on tags corresponding to molecular function or taxonomic information. Similar to results in NLP, scaling protein language models to very large sizes seems promising for protein sequences (Hesslow *et al.* 2022).

Transformer-based architectures (Vaswani *et al.* 2017), which we use in the present work for sequence-to-sequence prediction, have also been used, e.g. for creating generic embeddings trained on almost all known protein sequences (Rives *et al.* 2021), the prediction of mutational effects (Meier *et al.* 2021), protein interaction prediction and protein family classification (Nambiar *et al.* 2020), MSA-based language modeling (Rao *et al.* 2021), protein contact prediction (Zhang *et al.* 2021), inverse folding (Hsu *et al.* 2022, McPartlon *et al.* 2022), and have been at the core of recent breakthroughs in protein structure prediction (Jumper *et al.* 2021).

Recently, specific tasks have been cast as sequence-to-sequence translation problems using Transformers, similar to our approach. This includes, e.g. the generation of drug molecules given a protein sequence the molecule should interact with (Grechishnikova 2021) and the generation of short signal-peptides guiding the secretion of industrial enzymes, given the amino acid sequence of the enzymes (Wu *et al.* 2020).

Finally, non-neural network models borrowed from statistical mechanics have been extensively used in the context of sequence generation, e.g. generalized Potts models, a particular form of Markov Random Field (Figliuzzi *et al.* 2018). This type of model can be used for generating sequences using MCMC strategies, albeit with a significant computational cost. Relevant approximation strategies are, e.g. the recently introduced autoregressive (shallow) variants (Trinquier *et al.* 2021), which show a similar performance to Potts models but are computationally more efficient.

## 3 Data and methods

### 3.1 Dataset

Our data consist of 27 pMSAs containing domain sequence pairs that are part of the same multidomain proteins, taken from Muscat *et al.* (2020). The dataset contains only domain pairs which form a structural contact in at least one resolved PDB structure, making it likely that the two domains coevolve in order to maintain compatibility. We also extended our work to protein–protein interaction (PPI) by analyzing the dataset of histidine kinases and response regulators (HK-RR), which form the core of bacterial two-component signal transduction systems. In the case of PPI, we consider one protein as the context of the other, even if being on different proteins. HK-RR are a good case for our framework, since there are large and well-studied pMSA available (Anishchenko *et al.* 2017). Each dataset is comprised of *M* rows corresponding to *M* sequence pairs, where *M* depends on the dataset and ranges from a few hundred to more than 15 000, see Supplementary Appendix Section A for a summary of the datasets used. The sequences are already aligned using standard bioinformatics tools (Finn *et al.* 2011), which means that sequences belonging to the same domain family have the same length. Each row *l* in a dataset represents a pair of domain sequences $B^{l}$ and $A^{l}$, which are part of the same protein (or form an interacting pair of proteins). Every sequence consists of symbols denoting either 1 of 20 amino acids or an alignment gap, making the total size of the vocabulary equal to 21.

The first sequence $B^{l}=(b_{1}^{l},\ldots ,b_{N_{in}}^{l})$ is called the source or input sequence and we denote its length by $N_{in}$. It is used as an input to predict the second sequence, called the target or output sequence, $A^{l}=(a_{1}^{l},\ldots ,a_{N_{\mathrm{out}}}^{l})$, which is of length $N_{\mathrm{out}}$. All source sequences ${\left\{B^{l}\right\}}_{l=1}^{M}$ in the pMSA are members of the same domain family, and all target sequences ${\left\{A^{l}\right\}}_{l=1}^{M}$ in the pMSA are members of the same domain family. Each dataset was randomly split into a training set (70%) and a validation set (15%). The last 15% were kept as a testing set in order to be able to optimize hyperparameters for every domain, but we did not use it in the experiments shown in this work. Since sequences in an MSA are related to each other due to phylogeny, the validation set might contain sequences that are nearly identical to some sequences in the training set. We therefore further divided the validation set into two parts, one close to the training set and one far from it. This allows us to control for the effects of phylogeny on the performance metrics. This second splitting was made based on the median of the Hamming distance from the training set. The details for this subpartition are found in Supplementary Appendix Section A.

### 3.2 Performance metrics

#### 3.2.1 Log-likelihood and perplexity

An interesting property of autoregressive models, such as the Transformer or arDCA, is that they define a tractable probability distribution over the space of sequences. Contrary to, e.g. Potts Models and other energy-based models, we do not have to evaluate a global normalizing constant over the complete space of possible sequences. We can therefore calculate the log-likelihood of a sequence *A* given *B* as


(1) $$\text{log}\;P(A|B)=\sum_{i=1}^{N_{\mathrm{out}}}\;\text{log}\;(P(a_{i}|B,a_{1},\ldots ,a_{i-1})).$$

This is related to the cross-entropy, which we use as a loss during training, which we average over batches during training.


(2) $$L(A,B)=-\frac{\;\text{log}\;P(A|B)}{N_{\mathrm{out}}},$$

For assessing one aspect of the quality of our models, we use the closely related perplexity PP(A,B), which is a common quality metric for protein language models (Armenteros *et al.* 2020), and can be defined as


(3) $$PP(A,B)={\left(\prod_{i=1}^{N_{\mathrm{out}}}P(a_{i}|B,a_{1},\ldots ,a_{i-1})\right)}^{-1/N_{\mathrm{out}}}.$$

Below we show averages of the perplexity over the training and validation sets and use the notation $PP^{\mathrm{train}}$ and $PP^{\mathrm{val}}$ for these.

#### 3.2.2 Accuracy

While we use the perplexity as one metric for the quality of our model, it is not always easy to interpret: A high perplexity can result from a single wrong prediction with a high level of confidence. We therefore also use the accuracy A(A,B) for assessing our models. This measure takes the same input as the cross-entropy (the conditional probability for every position) and counts the fraction of times where the true amino acid is the one with the highest probability, leading to where V is the alphabet of symbols and *I* is an indicator function that is 1 if its argument is true, and 0 else. We define $A^{\mathrm{train}}$ and $A^{\mathrm{val}}$ as the average of the accuracy on the training and validation set.


(4) $$A(A,B)=\frac{1}{N_{\mathrm{out}}}\sum_{i=1}^{N_{\mathrm{out}}}I\left(a_{i}=\underset{\hat{a}\in V}{\mathrm{argmax}}\left[P(\hat{a}|B,a_{1},\ldots ,a_{i-1})\right]\right),$$

#### 3.2.3 Matching specificity

We expect the interaction between two domains to affect the probability distribution of the target sequence only marginally, with much of the variability in the distribution being explainable by constraints internal to the target sequence. As a consequence, a good performance in the quality measures defined above might be due to the decoder being a good language model of the target protein, possibly ignoring the input sequence altogether. We therefore also evaluate the specificity of the predicted target sequence given the source sequence.

Specificity is also related to the task of matching pairs of protein sequences, which is an active domain of research in bioinformatics (Bitbol *et al.* 2016, Gueudré *et al.* 2016, Szurmant and Weigt 2018). We implement this task by separating the source and target sequences in the validation pMSA, resulting in two separate MSAs with the same number of rows, one containing the source sequences and one the target sequences. We then shuffle the rows in the target MSA randomly and attempt to use our models to find the permutation of the target sequences that matches the original order. In order to create a matching based on a model, we calculate the log-likelihood of every combination of source and target sequences in the shuffled validation set and create a matching between source and target sequences based on the Hungarian algorithm (Kuhn 1955).

We then use the fraction of correctly matched pairs as an additional metric for the performance of our model, formally defining it as where $M_{\mathrm{val}}$ is the size of the validation set. Note that the difficulty of this task increases with the size of the validation set, since the expected fraction of correctly matched pairs using a random matching is $1/M_{\mathrm{val}}$.


(5) $$M^{\mathrm{val}}=\frac{\#\;\;\text{of correctly matched pairs in validation set}}{M_{\mathrm{val}}},$$

### 3.3 Transformers and baselines

We mainly used two Transformer models with different sizes, calling one the *shallow* and one the *large* model. The shallow Transformer consists of two layers with a single attention head, has an embedding dimension of $d_{\mathrm{model}}=55$, and a forward dimension of $d_{ff}=2048$. The large Transformer consists of three layers and has an embedding dimension of $d_{\mathrm{model}}=105$ with the same forward dimension and number of heads as the shallow transformer. Further details on their architectures can be found in Supplementary Appendix Section B.1. Both models are relatively small compared with Transformers trained on large protein sequence databases (cf. e.g. Hesslow *et al.* 2022, Lin *et al.* 2022). This can be explained in two ways. Firstly, when looking at one pair, the task is simpler as we only need to model a small fraction of the protein space where sequences can be aligned. Secondly, the smaller the number of training points gives rise to a complex overfitting problem that we analyze in Section 3.4 and Supplementary Appendix Section C.1. We compare their performance to the recently introduced shallow autoregressive model called arDCA (Trinquier *et al.* 2021) and a fine-tuned version of Rita L (Hesslow *et al.* 2022). While details on these methods can be found in Supplementary Appendix Section B.2, we note here that Rita was pretrained on a large corpus of unaligned, full-length sequences, which is a different setting from the pMSAs that we use for the Transformers. We, therefore, evaluated Rita only on unaligned, full-length sequences. For arDCA, which we train from scratch on pMSAs, there is no such mismatch and we can use it on the same pMSAs as the Transformers. For the datasets used in this work, the training time of the Transformer models ranges from less than an hour to about 1.5 days for the large Transformer on the largest dataset. The training was done using a single Nvidia V100 GPU. When using entropic regularization, which we will introduce in a later section, the training time increases significantly. In addition, we also trained a larger Transformer trained on nearly all the pairs. This Transformer has five heads, four layers, and a $d_{\mathrm{model}}=205$. The goal was to understand whether training on the joined dataset would enable transfer learning, or if, by mixing sequences from different families and alignments, it would make the task harder for the model. To enable a fair comparison we also replaced the <SOS> token with a token indicating the domain pair it was modeling. We need to give this hint to help the model know which family it has to generate. We held out three pairs of domains in order to check whether this joined Transformer was showing some transfer learning between families. We observed a loss of performance of approximately 3% in accuracy, 7% in matching, and 0.7 in perplexity. We concluded that a specialized Transformer for each pair, smaller and trainable in a few hours, is more suitable for the protein domain redesign task of this work. Complete results of the joined Transformer can be found in Supplementary Appendix Section B.4. We provide a table with training times in Supplementary Appendix Section B.5.

### 3.4 Entropic regularization

When experimenting with the large Transformer, we observed strong overfitting of the perplexity, especially when trained on smaller datasets. While this could be expected, we found that the matching performance was not following the same trend: While the perplexity started to degrade at some point during training, which is indicative of overfitting, the accuracy, and the matching performance were still increasing, see Supplementary Appendix Section C.1. While the shallow Transformer is less prone to overfitting, most likely due to its limited capacity, we found it necessary to introduce regularization for the large Transformer. We experimented with dropout and weight decay with limited success. While both schemes prevent overfitting in terms of perplexity, the matching performance and the accuracy dropped significantly. We show this effect in Supplementary Appendix Section C.1 for different training set sizes and regularization settings.

In order to find models with a good performance on perplexity, matching, and accuracy at the same time, we explored other regularization approaches.

In this section, we present an approach based on entropic regularization, where we enforce the probability of a target sequence *A* given a source sequence *B* to be similar to other sequences sampled from the model conditioned on *B*. This encourages the model to give similar weights to different possible interaction partners, even if there is only a single one present in the training set.

We therefore add a regularization term $\frac{1}{T}\sum_{l=1}^{T}R_{\mathrm{ent}}(A^{l},B^{l})$ to the loss, where *l* indexes the input sequence $B^{l}$ and the target sequence $A^{l}$ in the batch and *T* is the batch size. We sample *S* different target sequences for $B^{l}$ from the model. We denote the *k*th sampled sequence conditioned on $B^{l}$ as $A^{l,k}$. We sample using a Gumbel-Softmax distribution (Jang *et al.* 2016), which enables back-propagation through the sampling step. For computational efficiency, we sample every amino acid in $A^{l,k}$ conditioning on the preceding amino acids of the true $A^{l}$. Then we evaluate the log-likelihoods $R^{l,k}$ of the target sequence $A^{l,k}$ given $B^{l}$ and the log-likelihood of the true pair $R^{l}$,


(6) $$\begin{array}{c} R^{l,k}=\text{log}\;P(A^{l,k}|B^{l})\;\;\;\forall k=1,\ldots ,S\\ R^{l}=\text{log}\;P(A^{l}|B^{l}). \end{array}$$

We then use these quantities as the input for a log-softmax operation, resulting in


$$\begin{array}{c} R_{\mathrm{ent}}(A^{l},B^{l})=\text{log}\;P(A^{l}|B^{l})\\ -\text{log}\;\left(P(A^{l}|B^{l})+\sum_{k=1}^{S}P(A^{l,k}|B^{l})\right). \end{array}$$

**Figure 2.**
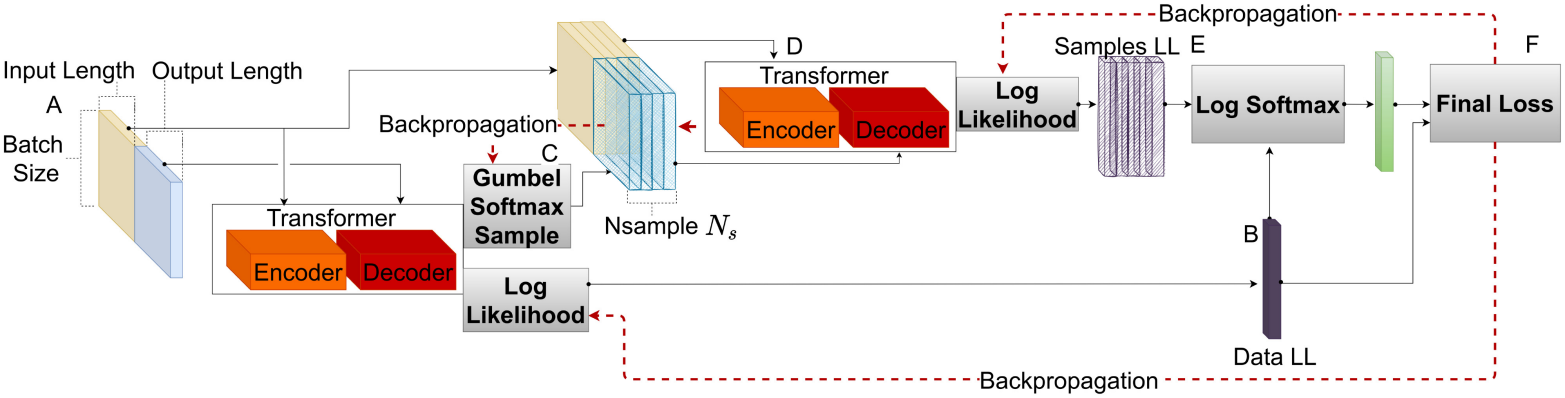
Diagram explaining training with entropic regularization: Panel (A) corresponds to the training batch, with yellow being the input protein sequences and blue the output protein sequences. At (B), the batch is sent to the Transformer and the log-likelihood Data LL is computed. At (C), $N_{s}$ output protein sequences are sampled from the Transformer for every input protein sequence using the Gumbel softmax operation. At (D), we evaluate the log-likelihood of the sequences sampled at (C) and call it Samples LL. At (E), we measure how well the loglikelihood separates the training sequences from the sampled sequences using a logarithmic softmax, creating an additional loss term; At (F), the losses calculated at (E) and (B) are combined. Gray boxes correspond to operations that do not include learnable parameters.

This term is multiplied by a factor α>0 to regulate its strength and *added* to the loss function, meaning that we aim to minimize it. This enforces similar probabilities for the true target sequence $A^{l}$ and the sampled target sequences $A^{l,k}$, conditioned on $B^{l}$. A diagram summarizing the regularization approach can be found in Fig. 2. A closer look reveals that it is a form of entropic regularization, maximizing the conditional Rényi entropy of order 2, see Supplementary Appendix Section C.2.

## 4 Results

### 4.1 Performance gain from context sequence

We first tested whether the input sequences had any effect on the perplexity of the target sequence. As already mentioned before, this is not self-evident, since the Transformer decoder itself could be a good model for the target sequence distribution without taking the input into account. We, therefore, trained two shallow Transformer models, one with the normal training set and one where we randomly shuffled the pairing between input and output sequences. We then evaluated the models on the normal validation sets, without shuffling. We expect that if the model trained on the normal training set exploits the information in the inputs when predicting the output, it should show a considerably lower perplexity than the model trained on a shuffled dataset.

**Figure 3.**
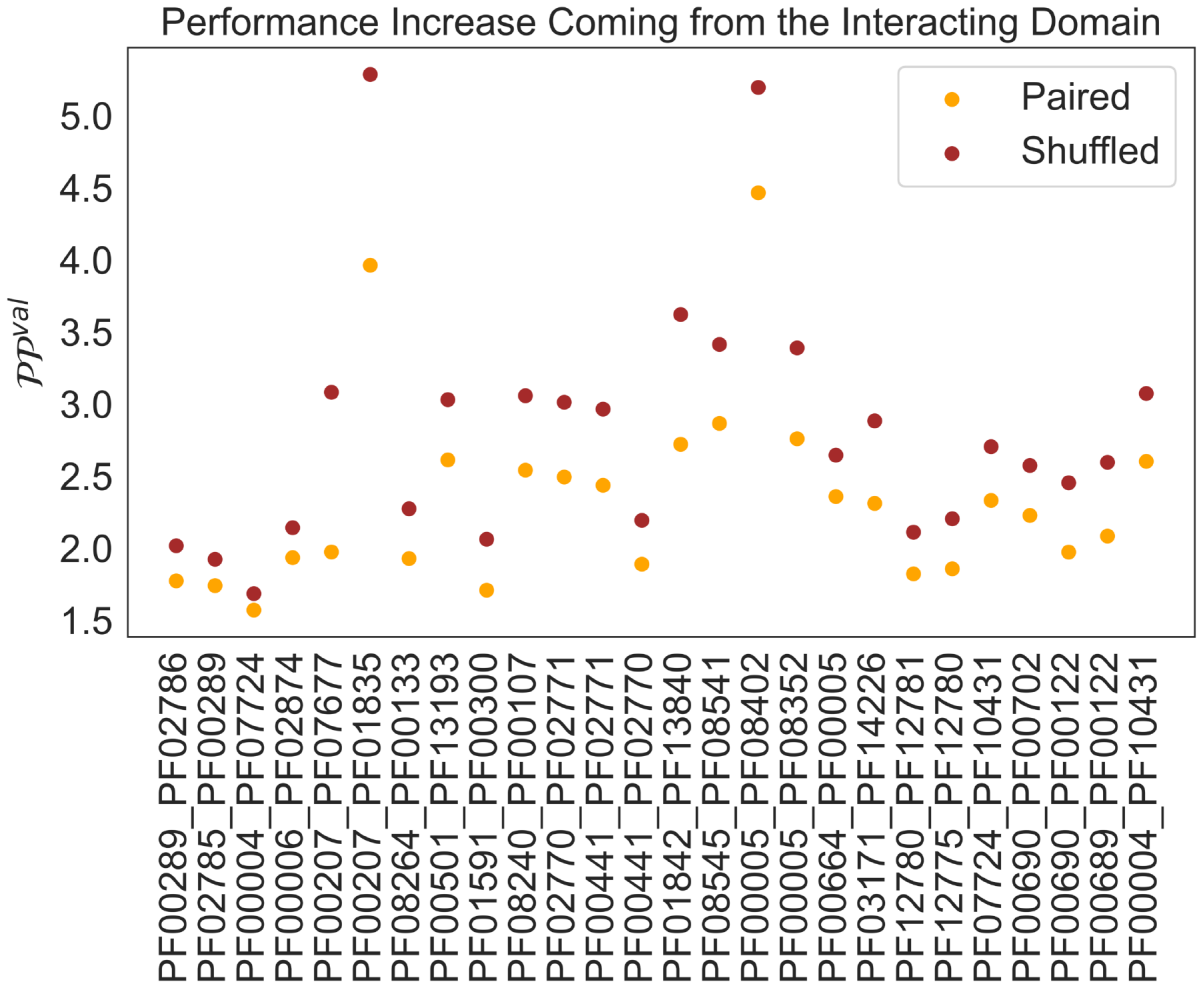
Performance (lower better) increases when taking domain sequence in context into account. We plot the perplexity $PP^{\mathrm{val}}$ for target sequences on the validation set, once for a shallow Transformer trained with the true pairings (Paired) and once for a shallow Transformer trained with shuffled pairs in the training set. (“Paired” is always below “Shuffled” with an average difference of 0.48).

We show the results of these experiments in Fig. 3. As can be seen, the models trained on the normal dataset have a significantly lower perplexity than the models trained on a shuffled dataset. This corroborates and quantifies the idea that domain sequences that appear in the context of a second domain contain information that can be used for modeling the constraints on the sequence of the second domain. We note that the difference in the logarithm of the perplexity, which is equivalent to the cross entropy, can be seen as a rough estimate of the mutual information between the output and the input. When the input sequence is randomly chosen, there is no correlation between the input and the output, and the corresponding probability can be seen as the marginal probability of the output sequence. We can therefore write where *a* and *b* are paired sequences. On the left-hand side of the equation, the sum is on the complete sequence space whereas on the right-hand side, the sum is only over the sequences in the validation set.


(7) $$MI=\sum_{a,b}P(a,b)\;\text{log}\;\left(\frac{P(b|a)}{P(b)}\right)\approx \sum_{a,b\in V}\;\text{log}\;\left(P(b|a))–\text{log}(P(b)\right),$$

### 4.2 Results on performance metrics of the shallow Transformer

**Figure 4.**
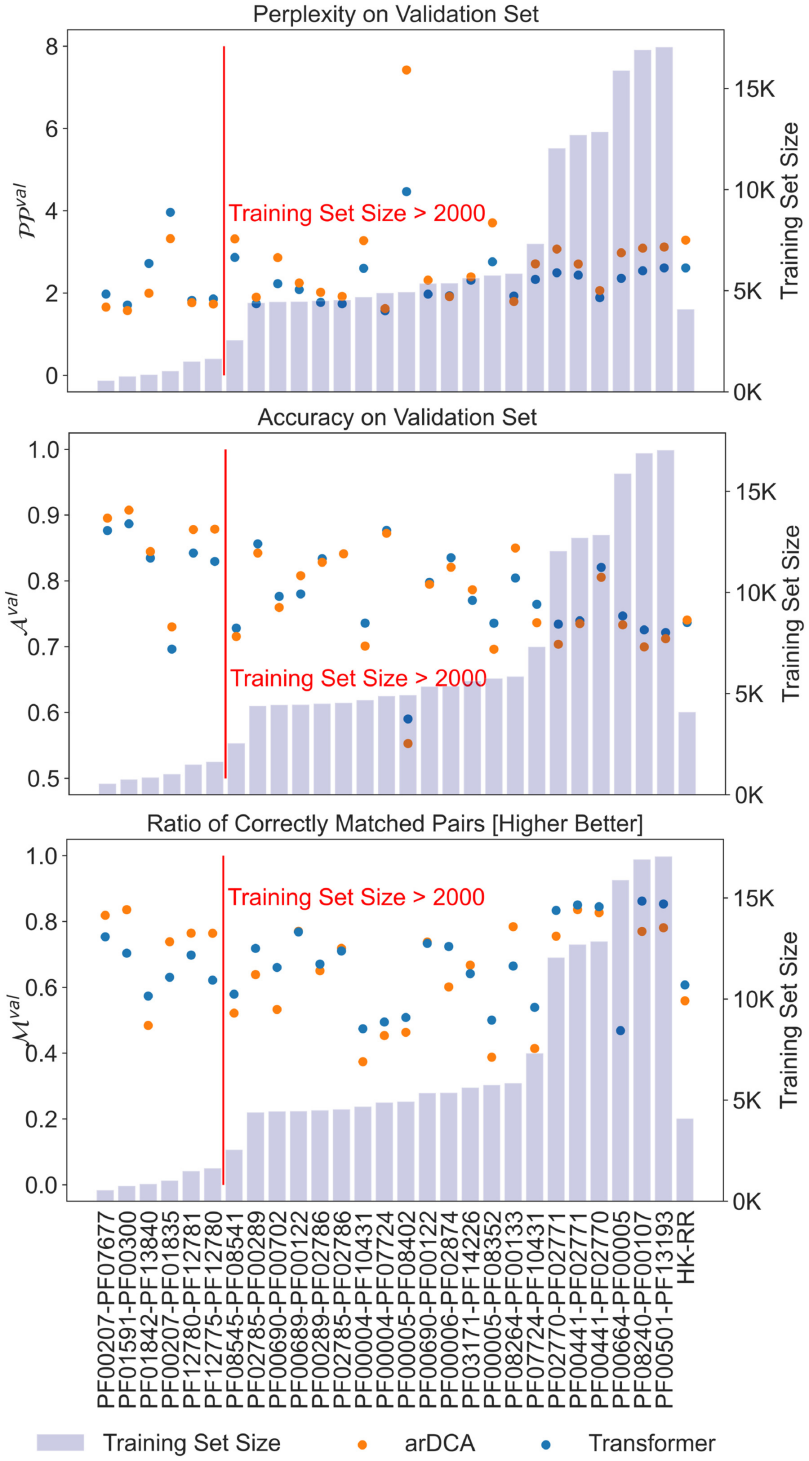
Perplexity $PP^{\mathrm{val}}$, accuracy $A^{\mathrm{val}}$, and the matching performance $M^{\mathrm{val}}$ for shallow Transformers and arDCA on validation set. The families pairs are ordered by training set size, followed by the PPI pair, HK-RR. Perplexity below 2000 examples: arDCA is always below Transformer with an average difference of 0.33. Perplexity above 2000 examples: Transformer is below arDCA in 91% of cases with an average difference of 0.48. Accuracy below 2000 examples: arDCA is always above Transformer with an average difference of 0.02. Accuracy above 2000 examples: Transformer is below arDCA in 81% of cases with an average difference of 0.01. Matching fraction below 2000 examples: arDCA is above Transformer in 83% of cases with an average difference of 0.07. Matching fraction above 2000 examples: Transformer is below arDCA in 77% of cases with an average difference of 0.05.

We next compared the shallow Transformer models to the arDCA baseline. Shallow Transformers outperform arDCA on nearly all datasets above a certain training set size in terms of perplexity, accuracy, and matching with a large margin, as can be seen in Fig. 4. We note here that the Transformer models, both shallow and large, have *fewer* parameters than arDCA for every family size we tried: The number of parameters in the Transformer models is independent of the length of the input and target sequences, while the number of parameters in the arDCA models scales quadratically with the concatenated input length.

The best performance is achieved for the families with the largest training sets, indicating that the performance of the Transformer might further increase with increasing training set size. Comparing the fraction of correctly matched paired between pairs is not straight forward. The matching task gets harder with the size of the validation set. For each input sequence, there is only one correct partner, which has to be identified in between all other proteins in the validation set. We repeated the calculation of the matching performance on subsampled versions of the validation set in order to obtain a better understanding of the matching performance of the model, see Supplementary Appendix Section F.

We also considered the possibility of using a large language model trained on protein sequences for our task. To this end, we tested and fine-tuned Rita L (Hesslow *et al.* 2022), a 680-M parameters model trained for predicting the next amino acid in a sequence.

Given that Rita is trained on full-length unaligned sequences, we used RITA also on full-length unaligned sequences, comparing the metrics only on match positions as predicted by the Pfam HMM of the corresponding domain family (excluding gaps and inserts).

**Figure 5.**
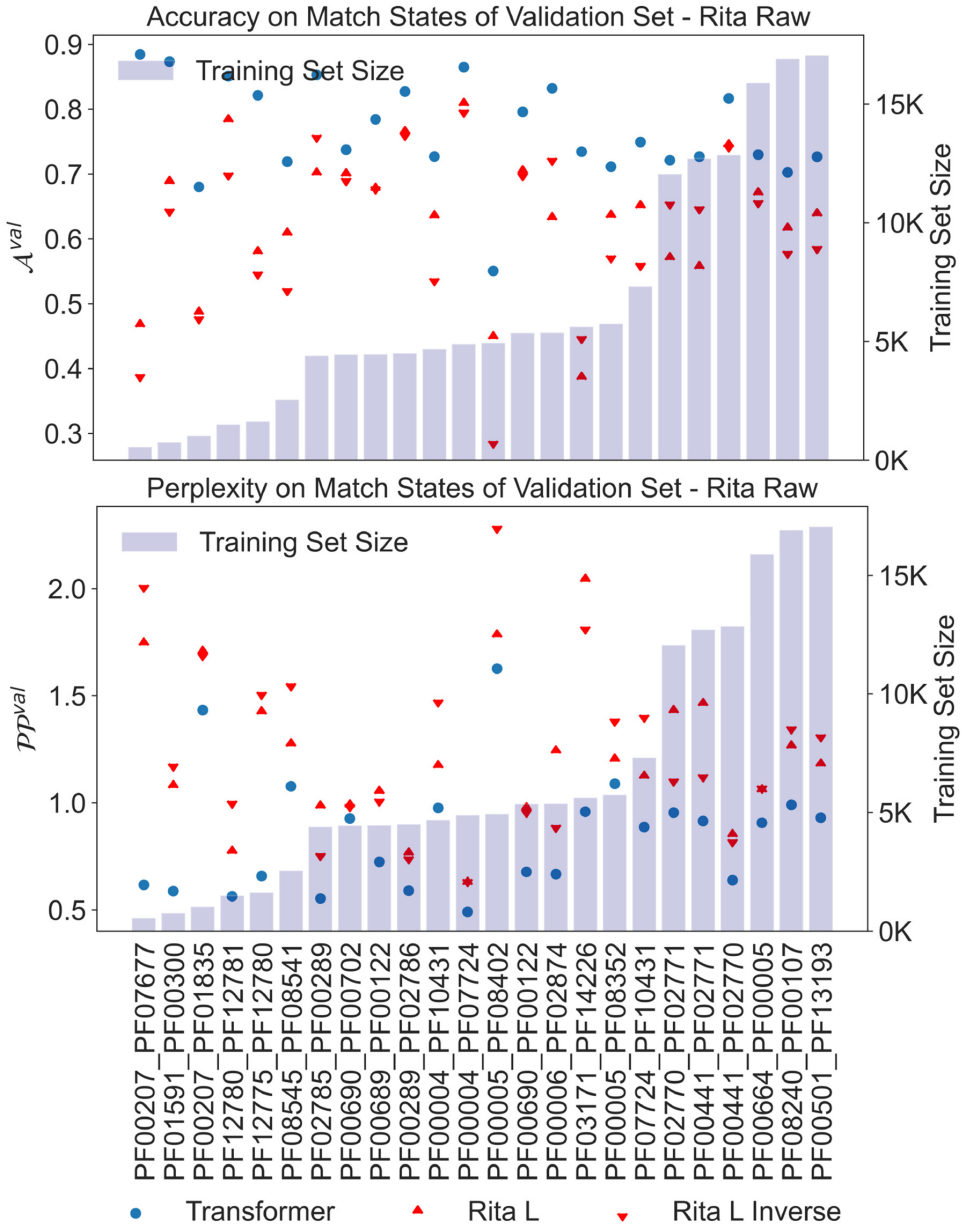
Perplexity $PP^{\mathrm{val}}$ and accuracy $A^{\mathrm{val}}$ for shallow Transformers and RITA L on validation set. The families are ordered by training set size. RITA L inverse refers to RITA when given the inverse sequence as input (RITA is trained on both original and inverse sequences).

According to our metrics, a large language model like RITA seems to underperform our family-specific Transformer by a large margin see Fig. 5. We, therefore, fine-tuned RITA for each of the domain–domain pairs. We should also note that RITA is only able to model single, full-length, proteins, meaning that it cannot be applied to the PPI task of HK-RR.

**Figure 6.**
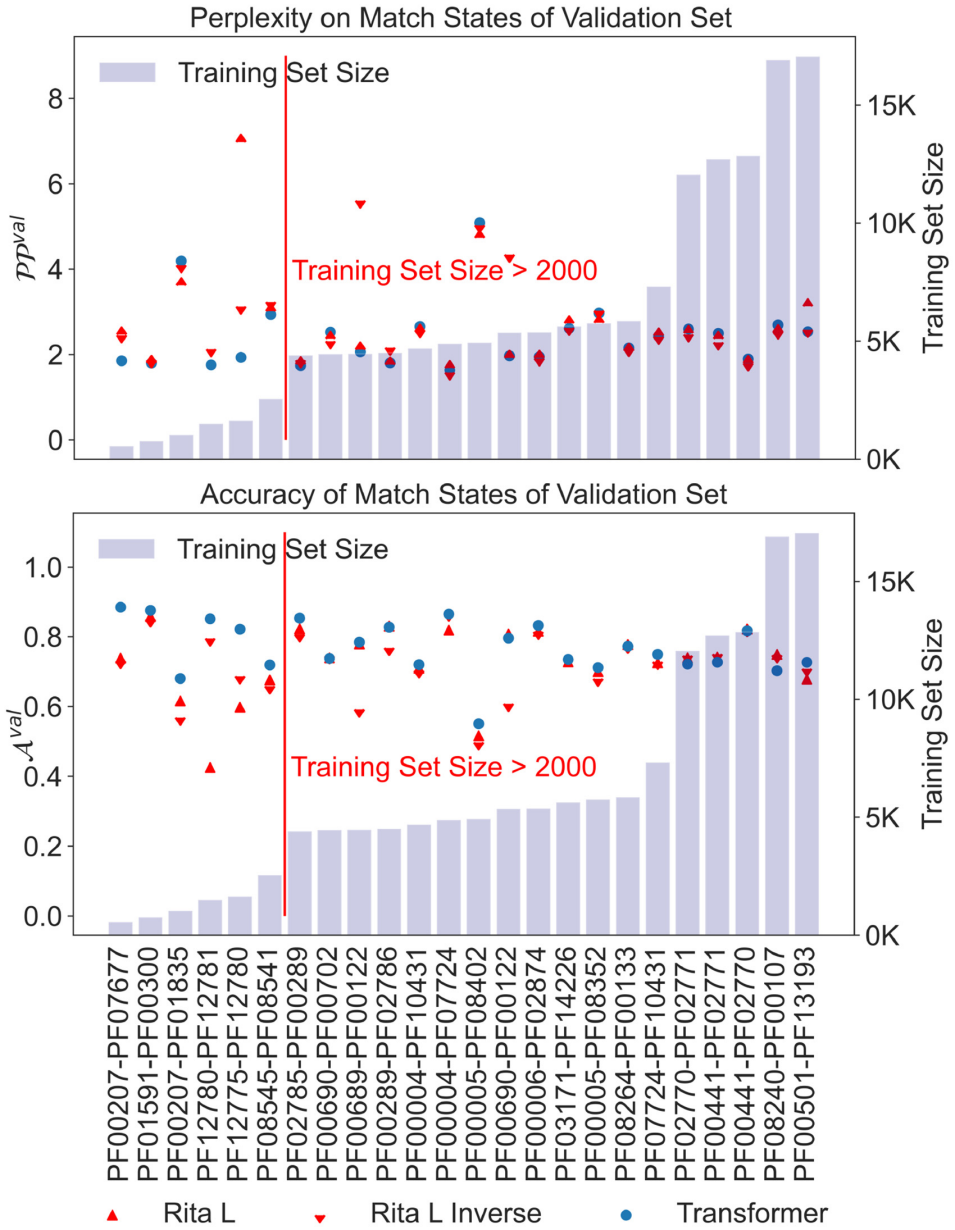
Perplexity $PP^{\mathrm{val}}$, accuracy $A^{\mathrm{val}}$, and the matching performance $M^{\mathrm{val}}$ for shallow Transformers and fine-tuned RITA L on the validation set. The families are ordered by training set size. RITA L inverse refers to Rita when given the inverse sequence as input (Rita is trained on both original and inverse sequences).

The details of the fine-tuning can be found in Supplementary Appendix Section B.3. The results are comparable with the domain-to-domain Transformer model, see Fig. 6, with the Transformer having a slightly higher accuracy. We note that Rita models are trained on Uniref100 and we suspect that most of the sequences in our validation set are in the training set of Rita, so this comparison is likely biased in favor of Rita.

### 4.3 Performance of the entropic regularization

**Figure 7.**
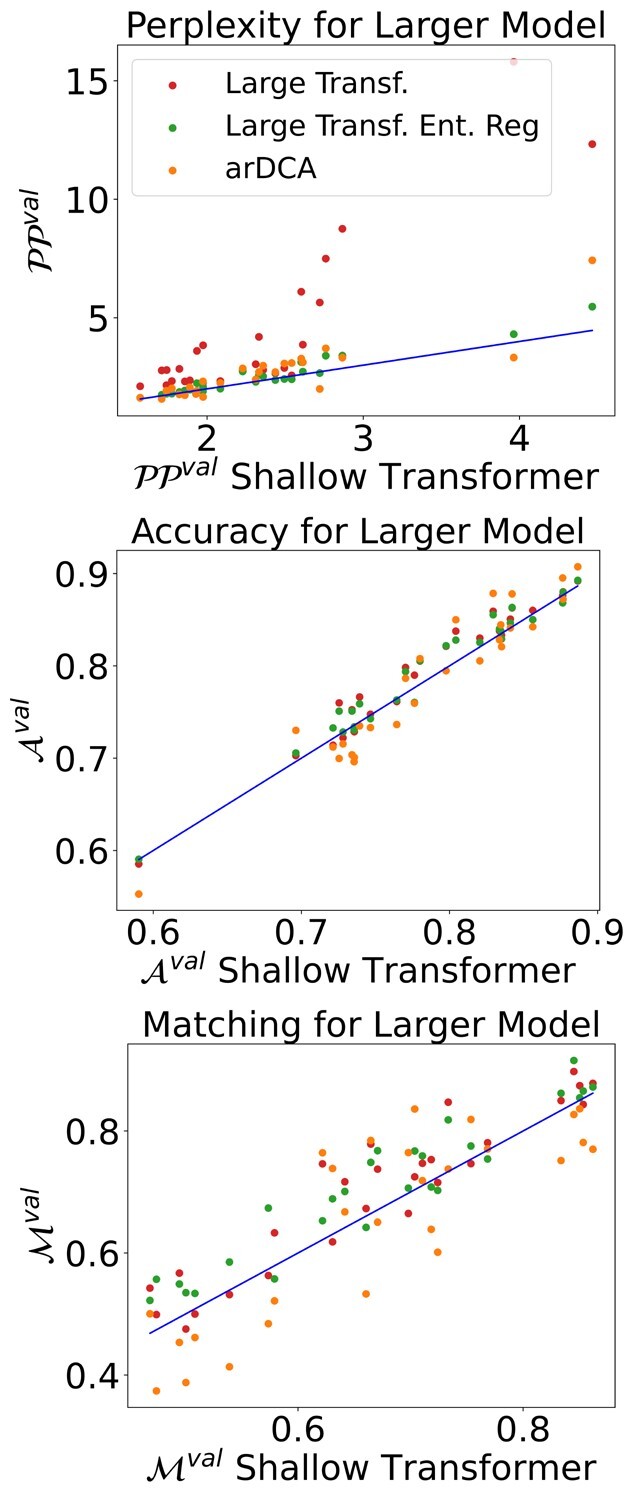
Comparison of the performance of the large Transformer without regularization (red), with entropic regularization (green) and arDCA (orange), and the shallow Transformer. The blue lines have a slope 1.

We performed a set of experiments on the 27 datasets in order to see if this type of regularization improves the performance. We retrained the large Transformer with and without the entropic regularization. We used S=5 and α=0.7 for the experiments. The results can be seen in Fig. 7, where we plot the performance of the shallow Transformer against the performance of the large Transformer for different regularization schemes and arDCA. The details of the training, models, and of performance for every family can be found in Supplementary Appendix Section C.2.1. The large Transformer outperforms the shallow Transformer in terms of accuracy and matching both with and without regularization, indicating that the large Transformer extracted more useful information from the training set. However, the large Transformer without regularization has a significantly higher perplexity on the validation set, indicating overfitting. Adding the entropic regularization leads to a good performance of the large Transformer in all metrics.

We also performed a systematic comparison of the entropic regularization scheme with standard weight decay, testing different weight decay values for the large Transformer. The details of the experiments and the results for every family can be found in Supplementary Appendix Section C.3.

### 4.4 Generalization and phylogeny

One specific characteristic of protein sequences, compared with data in NLP, is the structure of the data. The sequences in our datasets have a phylogenetic bias, visible as clusters of similar sequences in the data, that are simply explained by a close common ancestor. This bias makes a random split unsuitable since the test set will contain sequences that are very similar to some sequences in the training set. We, therefore, evaluate our model on different subsets of the test set, which are selected based on the similarity to the training set.

**Figure 8.**
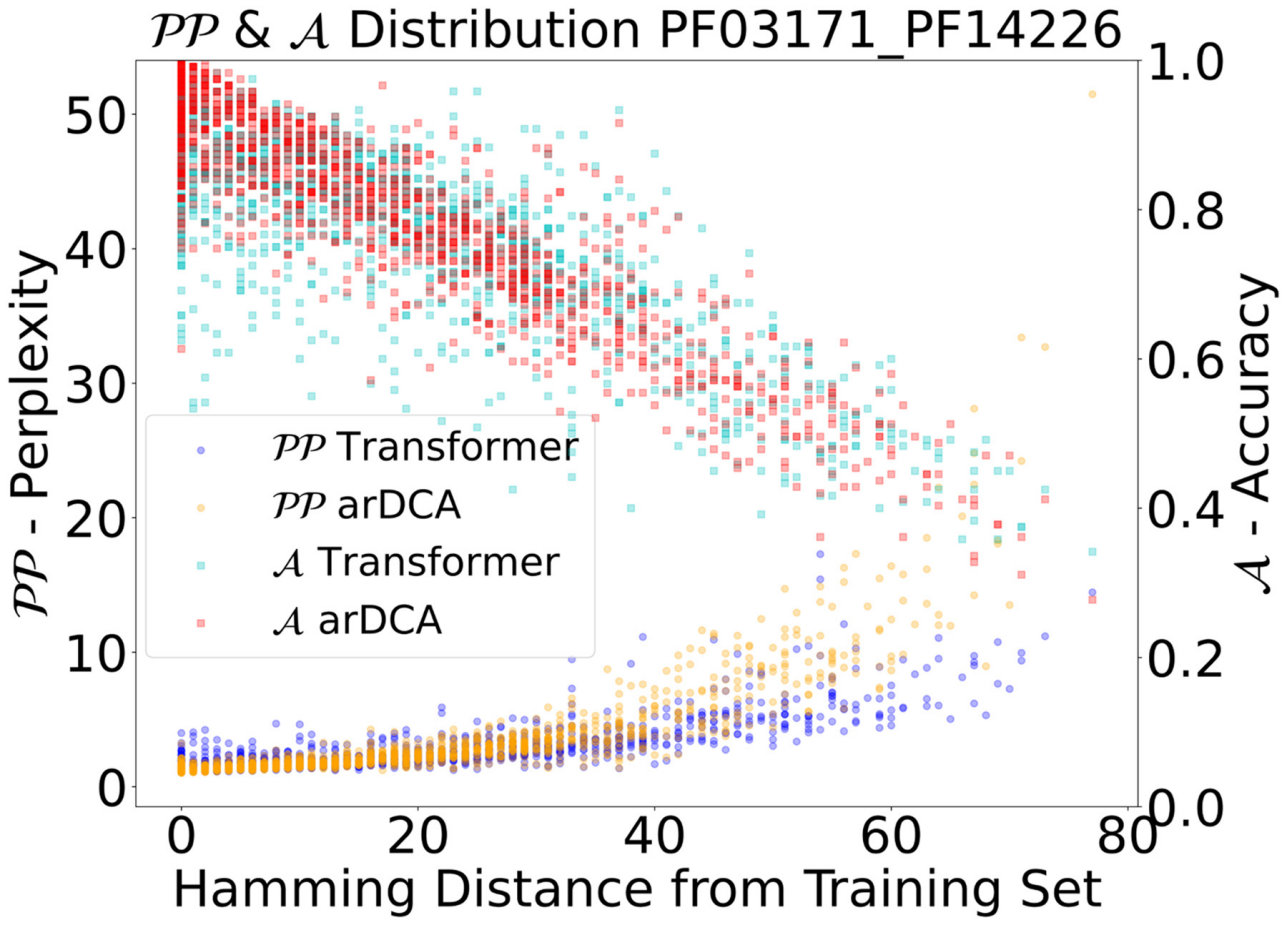
Perplexity (*lower* is better) and accuracy (*higher* is better) for every sequence of the validation set in dependence on the distance of the sequence from the training set. The distance of a sequence to the training set is the Hamming distance to the closest sequence in the training set.

We show the perplexity on target sequences in the validation set in dependence of the distance from the training set in Fig. 8, where the distance of a sequence to the training set is the smallest Hamming distance from the sequence to any training sequence. Interestingly, it seems that the advantage in performance of Transformer models over arDCA is mostly due to sequences far away from the training set, indicating that Transformers generalize better in regions of sequence space far away from the training set. We also verified that this advantage holds for matching. To do so, we split the test set into the half closer and the half further from the training set. When matching the pairs, we look at the performance on these two subdatasets. The details of these results can be found in Supplementary Appendix Section E.1 for the shallow Transformer and at Supplementary Appendix Section C.3 for the large and regularized Transformer.

### 4.5 Structural information and generative properties

In this section, we show further results on the performance of the shallow Transformer.

**Figure 9.**
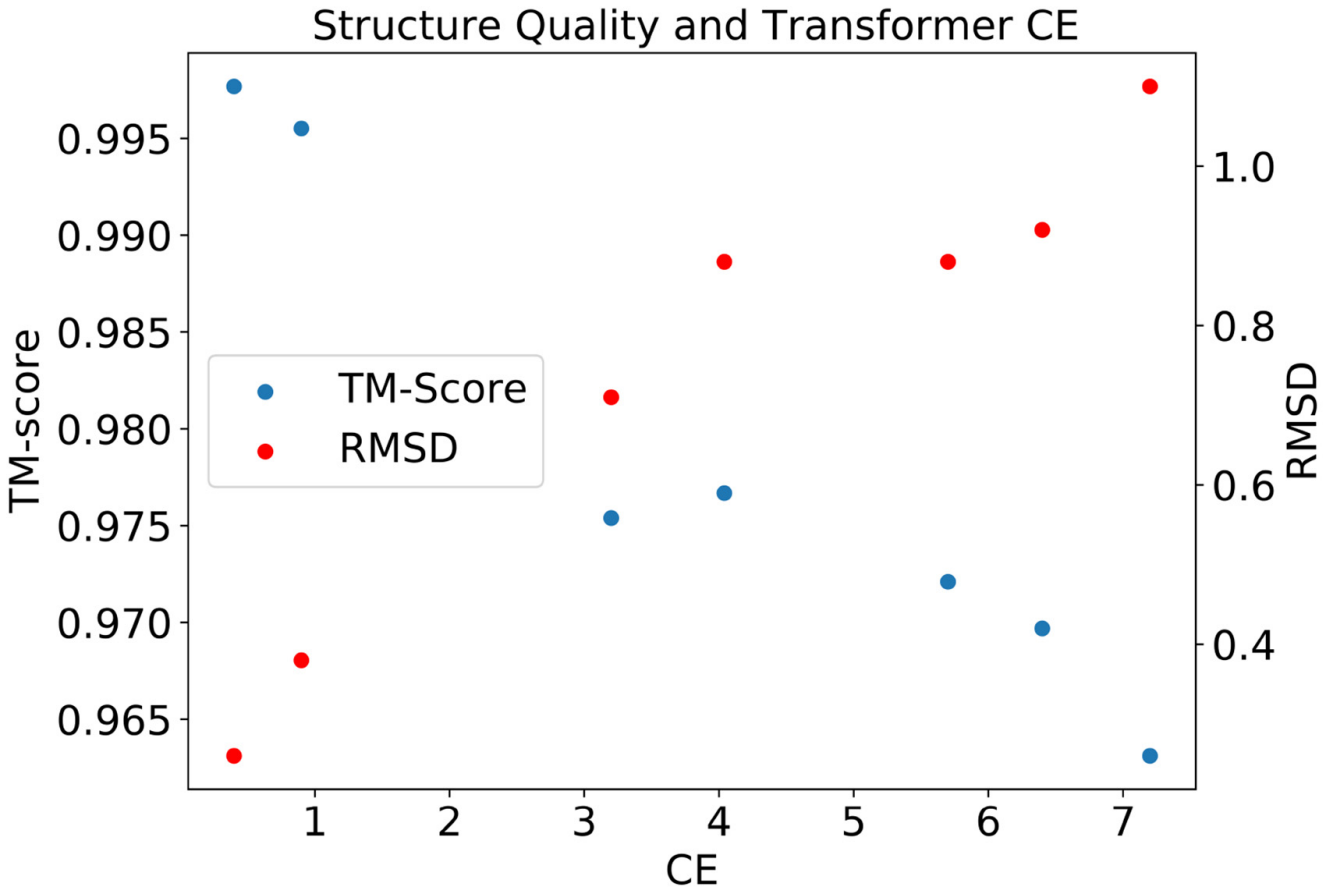
TM-scores and RMSD values when comparing the Alphafold-predicted structures of true sequences with Alphafold-predicted structures of sequences where single domains have been replaced with homologous natural sequences. The results are based on Q1H158, which contains domains PF00289 and PF02785, which are in contact in PDB 5ks8. Homologous PF00289 sequences are sampled from the validation set and inserted into the Q1H158 sequence, measuring the change in structural scores and in cross-entropy in the shallow Transformer model (abscissa).

We tested whether the target sequence distribution of trained Transformers is integrating structural information. To do so, we explored the correlation of our metrics with scores related to structure prediction when using Alphafold (Jumper *et al.* 2021). To this end, we selected the protein Q1H158 from the validation set, which contains the Pfam domains PF00289 and PF02785. We then replaced the domain PF00289 with homologous sequences from the validation set, keeping the rest of the Q1H158 sequence unmodified. The resulting sequences contain natural sequences for both domains but in a combination that does not exist in any known protein. We then used Alphafold to predict the structure of the original sequence and the modified sequence, comparing them using the TM-score and the RMSD on the two domains. We found these structural metrics to be well-correlated with the cross-entropy of the resampled PF00289 of the shallow Transformer conditioned on PF02785, see Fig. 9. We stress here that all domain sequences assessed here are natural sequences with presumably a high fitness, which makes it more likely that a higher cross-entropy for a pair is due to a decreased mutual incompatibility, reflected in the structural scores. We present results for more proteins in Supplementary Appendix Section D.1.

**Figure 10.**
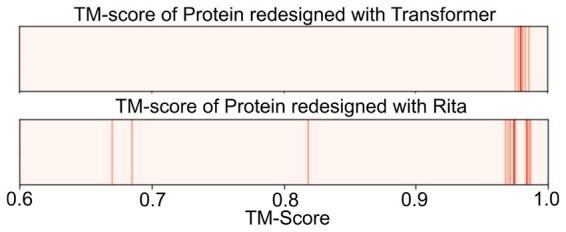
TM-scores comparing Alphafold structural predictions based on original and modified sequences of the protein Q1H158, which contains domains PF00289 and PF02785. The sequences are modified by resampling PF00289 from the shallow Transformer and Rita.

We next assessed the generative power of domain-to-domain Transformer models. To this end, we again used the protein Q1H158 as a test. We sampled novel PF00289 domain sequences conditioned on the PF02785 sequence found in Q1H158 using the shallow Transformer model. We then replaced the original domain sequence in Q1H158 with the sampled sequences and compared the structures predicted with Alphafold based on the original and modified sequences. For comparison, we also sampled sequences from Rita using beam search. We note that one reason for choosing Q1H158 is that the domain we want to redesign is at the end of the sequence, enabling a causal language model like RITA to sample the domain conditioned on the rest of the protein. We show the results in Fig. 10, where several sequences sampled with RITA have a significantly lower TM-score than sequences sampled from the Transformer. A closer analysis showed that some of these sequences did not contain a domain recognized by the Pfam HMM for family PF00289, indicating the fine-tuned Rita model did not always complete the sequence with the same domain as is found in the original sequence, as desired. While such alternative completions might very well correspond to a domain organization found in natural sequences, it shows that some care has to be taken when using unconditional language models for redesigning parts of a sequence, even if the model has been fine-tuned only with examples for the desired domain organization. On the other hand, the decoder of the shallow Transformer has been trained only for sampling the desired domain.

Finally, we looked at a method for unsupervised structural prediction called direct coupling analysis (DCA). We sampled for each input protein sequence of the training set eight target sequences, adding all sampled sequences together with the input sequences to a pMSA, which therefore contained natural sequences from the input domain concatenated to artificial sequences from the target domain. We then attempted to extract structural contacts between the two domains using plmDCA (Ekeberg *et al.* 2013), a popular method for prediction contacts. While the performance in contact prediction is worse than when using the natural target sequences directly, see Supplementary Appendix Section D.2, there is a strong signal with several correctly predicted contacts among the highest scoring residue pairs.

## 5 Discussion

In this work, we explored the use of Transformers for generating protein domain sequences while taking into account other domain sequences that are part of the same multidomain protein. We cast the problem as a translation task, which allowed us to directly use Transformers developed for translation between natural languages. We showed that this architecture is capable of outperforming state-of-the-art shallow autoregressive models in several metrics and explored a new regularization scheme optimized for our use case. Casting the task as a translation problem allowed us to use metrics like the matching performance for assessing the quality of the generative models.

Our work is placed at the intersection of two streams of research: There is a long history of building domain-specific generative models on aligned sequences for tasks like drug design or mutational effect prediction. More recently, however, large models based on Transformer architectures trained on all or nearly all unaligned protein sequences available have shown remarkable capabilities for capturing complex patterns in the data. Our work, on the other hand, solves a very generic sequence-to-sequence prediction task using smaller Transformer architectures, specialized for a family pair and using aligned sequences, which allows for domain-specific models. One limitation of our work is that we consider only a single domain as the context when predicting the sequence of an interacting domain, disregarding additional domains that might be present in the same protein. Conceptually, it would be interesting to enrich the context to multiple other domains or other biological information such as location or phylogeny.

An interesting question for further research is if we could observe a gain in performance due to transfer learning when training one model on a very large number of pairs. Given the successful extension to HK-RR, it would be interesting to apply this approach to other PPI problems, such as TCR-epitope binding.

## Supplementary Material

btad401_Supplementary_Data
