# Supplementary material for: Generating interacting protein sequences using domain-to-domain translation
Source: Bioinformatics. 2023 Jul 3;39(7):btad401. doi: 10.1093/bioinformatics/btad401 (PMC10329493; doi:10.1093/bioinformatics/btad401)
Supplement: btad401_Supplementary_Data [file btad401_supplementary_data.pdf]

Source Family	Target Family	N_{in}	N_{out}	M_{train}	M_{val}	d_{med}
PF00689	PF00122	184	223	4460	488	62
PF00289	PF02786	112	213	4502	495	35
PF02785	PF00289	110	112	4396	490	24
PF00004	PF07724	134	173	4886	1118	21
PF00006	PF02874	215	71	5370	464	15
PF02785	PF02786	110	213	4542	494	35
PF00207	PF07677	94	94	552	138	10
PF00207	PF01835	94	98	1027	241	43
PF08264	PF00133	154	604	5846	501	110
PF00501	PF13193	425	76	17050	498	120
PF01591	PF00300	225	196	760	189	31
PF08240	PF00107	111	132	16903	500	38
PF02770	PF02771	99	115	12048	499	33
PF00441	PF02771	152	115	12703	501	38
PF01842	PF13840	69	67	851	190	19
PF08545	PF08541	82	92	2548	556	35
PF00441	PF02770	152	99	12850	497	26
PF00005	PF08402	139	78	4933	1066	49
PF00005	PF08352	139	67	5751	1243	40
PF00664	PF00005	276	139	15889	3473	106
PF03171	PF14226	103	120	5624	1317	29
PF12780	PF12781	270	222	1498	361	37
PF12775	PF12780	274	270	1632	386	39
PF07724	PF10431	173	83	7315	1649	25
PF00690	PF00702	71	212	4450	486	63
PF00690	PF00122	71	223	5356	484	37
PF00004	PF10431	134	83	4685	1088	25
HK	RR	64	112	4086	1021	33

Table 1. List of the pairs of domains used in this dataset. N_{in} and N_{out} are the length of the input domain and the target domain. M_{train} and M_{val} and the size of the training and validation dataset.

Appendix for: Generating Interacting Protein Sequences using Domain-to-Domain Translation

Appendix A Datasets

In this section, we give details about the 27 family pairs used to measure the performance of the different models. The quantities N_{in} and N_{out} are the domain length of the source family and the target family, M_{train} and M_{val} the size of the training set and validation set and d_{med} is the median distance of a sequence in the validation set to the training set. This distance was used as a cutoff for distinguishing the matching performance for sequences close or far from the training set, which are denoted by $\mathcal{M}_{Close}$ and $\mathcal{M}_{Far}$.

Appendix B Methods and Models

B.1 Transformer

The translation model we use is matching closely the original Transformer model from Ref. (Vaswani *et al.*, 2017), featuring an encoder-decoder architecture. While we refer to this work for more details, we review here the key components. Sequences from the source family are encoded by the encoder and used as the input for the decoder. The source sequence is processed through alternating blocks of self-attention and linear layers. The same is done for the already translated part of the target sequence, while the part of the target sequences not yet decoded is masked. Typical vocabulary sizes in NLP are in the order of 10^4 to 10^5, while in our case we have a vocabulary $\mathcal{V}$ is composed of 21 tokens, corresponding to 20 amino acids and an alignment gap symbol.

The input embedding is composed of two parts, one for the amino acid identity and one for the position in the sequence. We learn a dictionary W, mapping each of the 21 symbols to a vector of dimension d_{model}. The sequence position is embedded as a vector PE, calculated as

$$\begin{aligned} PE_{(i,2k)} &= \sin\left(\frac{i}{1000^{\frac{2k}{d_{model}}}}\right) \\ PE_{(i,2k+1)} &= \cos\left(\frac{i}{1000^{\frac{2k}{d_{model}}}}\right) \end{aligned}, \tag{8}$$

where i is the position in the sequence and k is the dimension in the embedding vector. The embedding of a sequence is then taken as the sum of the amino acid and positional embeddings. The embedded amino acid sequences are then passed to the encoder, mapping them to a latent representation $z = (z_1, \ldots, z_n)$. This latent representation is then passed to the decoder that predicts the interaction partner sequence.

The decoder implements an auto-regressive distribution

$$P(a_i|z, a_{<i}), \tag{9}$$

defining the probability of the ith amino acid in the interaction partner sequence given the preceding amino acids in the interaction partner $a_{<i}$ and the hidden representation z of the input sequence B. During training, we use the true amino acids for $a_{<i}$, while during sampling we sample the sequence A sequentially.

Attention Mechanism Both the encoder and decoder use self-attention mechanisms and the decoder also the cross-attention mechanism.

Following (Vaswani *et al.*, 2017), we define the attention operation as

$$Attention\,(Q, K, V) = softmax\left(\frac{QK^T}{\sqrt{d_{model}}}\right)V, \tag{10}$$

where Q is the query matrix, K is the key matrix, V is the value matrix and d_{model} is the dimension of the keys, which we will define below.

For each element of the query Q we compute its similarity with the different values of the keys K. This yields weights used to compute a weighted average of the value V.

The output of the ith attention head, called $head_i$, is calculated as

$$head_i = Attention\left(QW_i^Q, KW_i^K, VW_i^V\right) \tag{11}$$

We then linearly combine the different heads, resulting in

$$Multihead\,(Q, K, V) = (head_1, \ldots, head_h)\,W^O. \tag{12}$$

In the self-attention layers, the keys, queries, and values are calculated from the same input. In this case, $K = Q = V$.

In cross attention, the keys and values are based on z and the queries on the intermediate decoder representations. In the results presented in this paper, we only used models with a single head.

Transformer Architecture The complete architecture is represented in Fig. B.1 and is based on stacking encoder and decoder blocks in the two parts. At the end of these blocks, linear with dimension d_{ff} and residual connections to the input of the blocks are added.

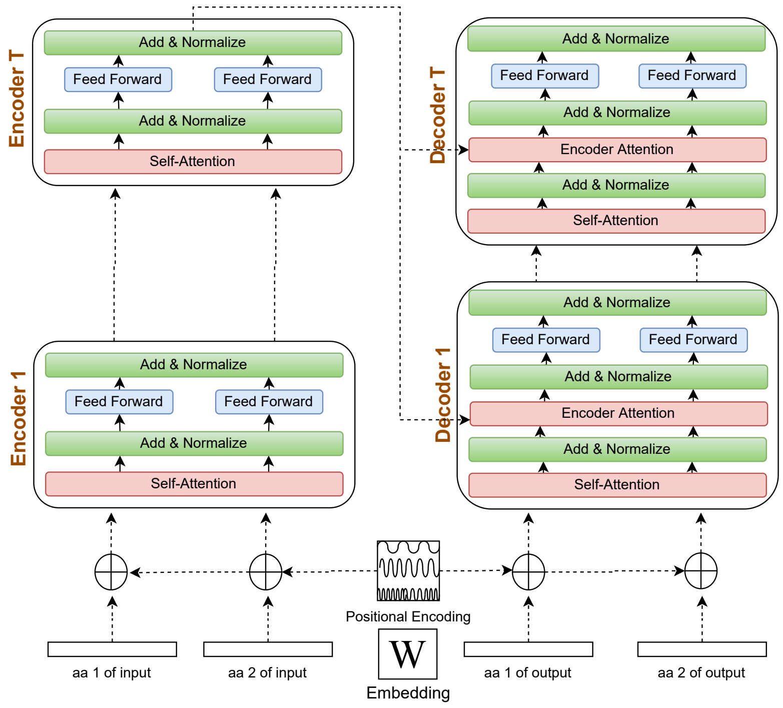

Fig. B.1. Transformer architecture for an input and output protein of length 2 and T layers: W is an embedding matrix, matching each amino acid to a vector of size e. The architecture here represents proteins of length 2 for simplicity, but the encoder and decoder can handle inputs of arbitrary length. The positional encoding uses sine functions of different frequencies to generate an embedding of the protein position.

The code is based on the PyTorch implementation of the Transformer https://github.com/pytorch/pytorch.

B.2 arDCA Baseline

As a baseline, we use the recently introduced arDCA (Trinquier *et al.*, 2021), which is an efficient autoregressive model for protein sequences. or an amino acid sequence $A = (a_i, ..., a_N)$ of length N, arDCA defines the conditional probability $P(a_i|a_{i-1}, ..., a_1)$ as

$$P(a_i|a_{i-1}, ..., a_1) = \frac{\exp\left\{h_i(a_i) + \sum_{j=1}^{i-1} J_{ij}(a_i, a_j)\right\}}{z_i(a_{i-1}, ..., a_1)}\,, \tag{13}$$

with $z_i(a_{i-1}, ..., a_1) = \sum_{a_i} \exp\{h_i(a_i) + \sum_{j=1}^{i-1} J_{ij}(a_i, a_j)\}$ being a normalization factor. The parameters h depend on a single position and the amino acid found at that position and the parameters J on pairs of positions and the two amino acids found at that pair of positions.

The probability of a sequence can be computed using the decomposition

$$P(a_1, ..., a_L) = P(a_1) \cdot P(a_2|a_1) \cdots P(a_L|a_{L-1}, ..., a_1), \tag{14}$$

which is tractable. Training can be done using standard convex optimization methods.

For our purposes, we concatenate the source protein B and the target protein A into a single sequence during training. During evaluation, we just need the conditional probability $P(A|B)$, which we calculate using

$$P(a_1, ..., a_{N_{out}}|B) = P(a_1|B) \cdot P(a_2|B, a_1) \cdots P(a_{N_{out}}|B, a_{N_{out}-1}, ..., a_1)\,. \tag{15}$$

We also added an L2 regularization on the parameters h and J. During our experiments, we used the regularization parameters communicated by the authors ($\lambda_h = \lambda_J = 0.0001$).

B.3 RITA

Rita is a decoder-only Transformer without conditioning information. It means that it only uses the decoder part of B.1, where the encoder-decoder attention layer has been deleted. This defines a generic autoregressive model. We used the Rita L model composed of Large $680M$ parameters, a model dimension of 1536, and 24 layers. This huge model was then trained on the UniRef-100 database. Moreover, we should note that the training was done in both directions. This explains why we present both scores when evaluating the performance of Rita finetuned. For finetuning Rita we used a batch-size of 6 and the Adam optimizer on the full-length sequence of the proteins in our train-set. Sequences were passed in both directions. Every Validation loss was computed every 200 gradient updates, and the best-performing model was kept for the experiments shown in the paper.

Rita is a language model based on the decoder of the original Transformer model in Fig.B.1. This means it does not use encoder-decoder attention and implements a generic unconditioned autoregressive sequence model. In our experiments, we use Rita L, which has $680M$ parameters, a model dimension of 1536, and 24 layers. The model we used for finetuning was pre-trained on Uniref-100 predicting in both the natural and the reverse direction of the protein sequences. We used a batch-size of 6 and the Adam optimizer for finetuning on the full-length sequences (both directions) on our datasets. We calculated the loss on the validation every 200 gradient updates on a total of 4000 gradient updates. We used the best-performing model for the experiments shown in the paper. The number of steps to finetuned each model varies between families but usually stands around 1000, way before the end of our training. To make the loss comparable we only took into consideration the positions that were match state for the Pfam HMM of the domain.

B.4 Joined Transformer

Here we detail more the performance of training a single Transformer (the "joined Transformer") on all the pairs in order to see if we can benefit from transfer learning between pairs. To do so we joined all the datasets except 4 in one single dataset. The left-out pairs are used for evaluating if there is transfer learning to unseen pairs. To make the comparison fairer we replaced the <SOS> token with a specific token for the pair (the idea is to tell to the Transformer the task it has to perform). To make this model also compatible with unseen pairs (and unseen specific tokens) we replaced this token only 50% of the time in the training examples, 100% of the time in the validation sets of seen pairs, and never for the unseen pairs. The training takes significantly longer. We trained in parallel for two days on 12 GPU whereas the smaller models were trained on a single one for usually less than a day.

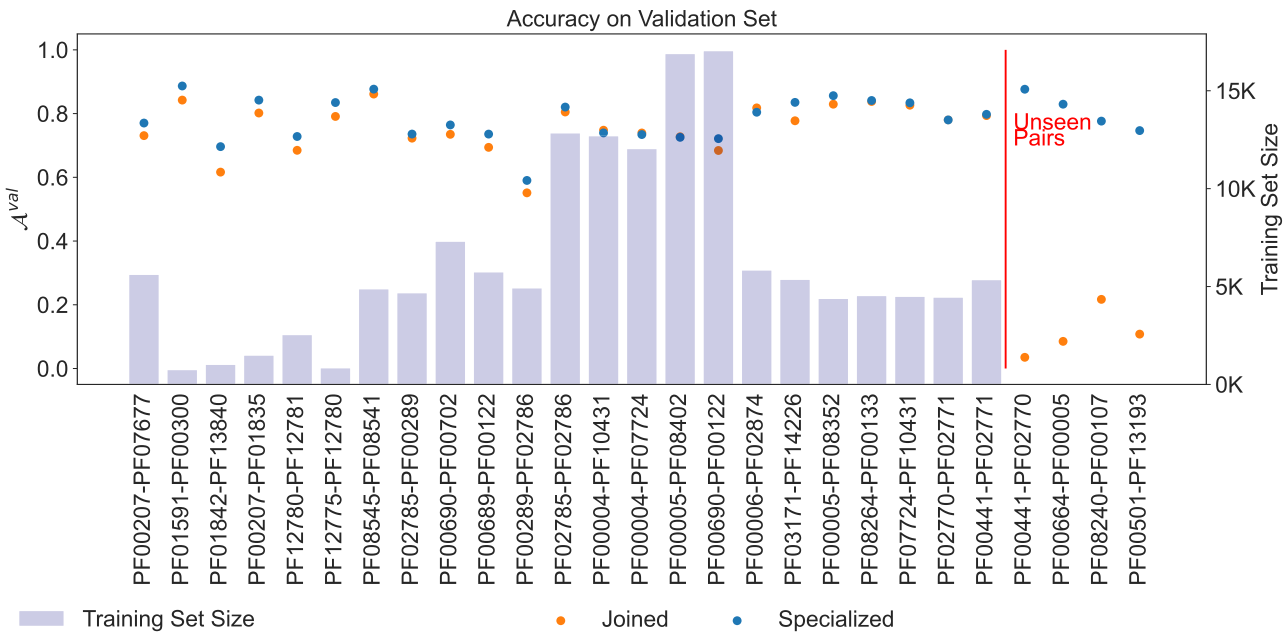

Fig. B.2. Accuracy $\mathcal{A}^{val}$ on the validation set for the shallow Transformer, and the Joined Transformer. The families are ordered by training set size.

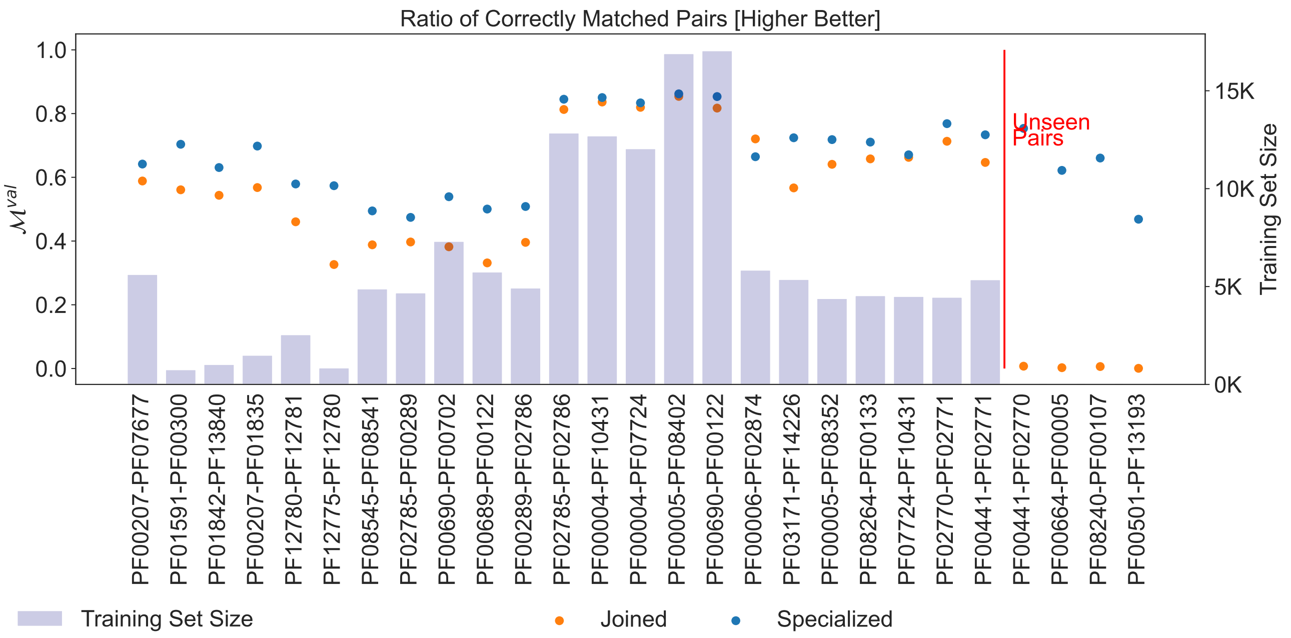

Fig. B.3. True positive rate for matching on the validation set for the shallow Transformer, and the Joined Transformer. The families are ordered by training set size.

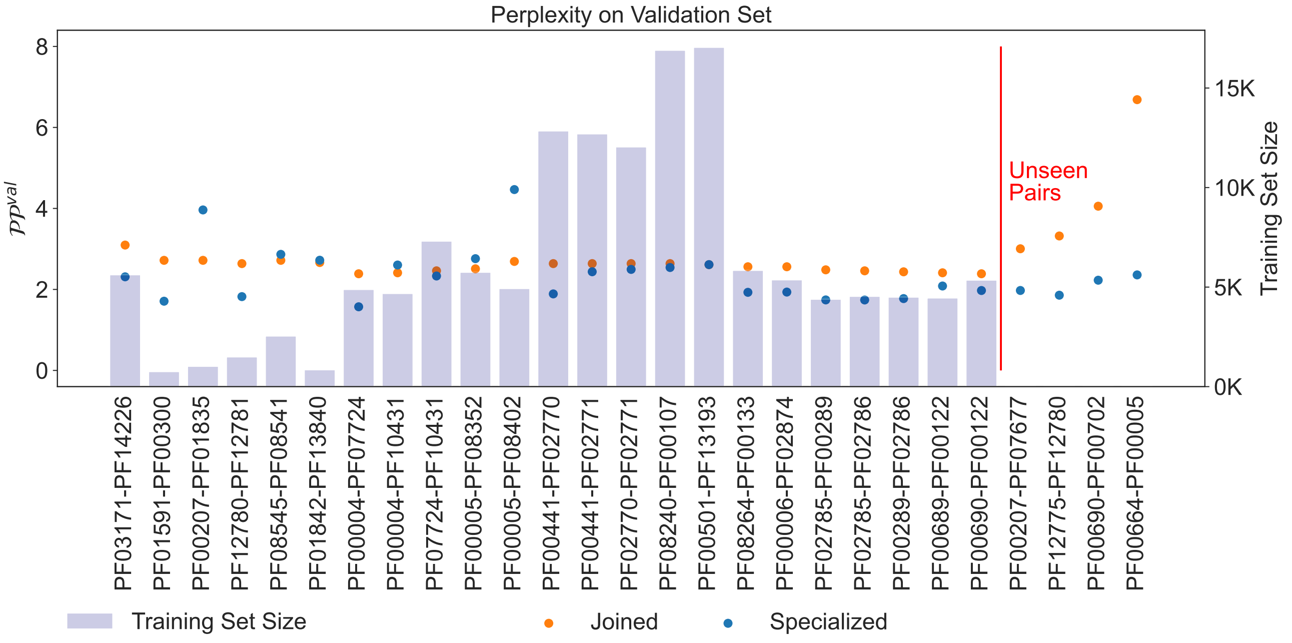

Fig. B.4. Perplexity $\mathcal{PP}^{val}$ for the shallow Transformer, the Joined Transformer on the validation set. The families are ordered by training set size.

The results seem to show very little transfer learning between pairs. Probably such an effect could only appear when training on thousands of pairs. Moreover, the spirit of this paper is intended to fit the line of work of domain-specific models like in Potts, VAE, RBM Hawkins-Hooker *et al.* (2021b); Tubiana *et al.* (2019); Russ *et al.* (2020). We intend to guide specific design task when one wants to redesign a specific domain/protein to increase its fitness for a desired task.

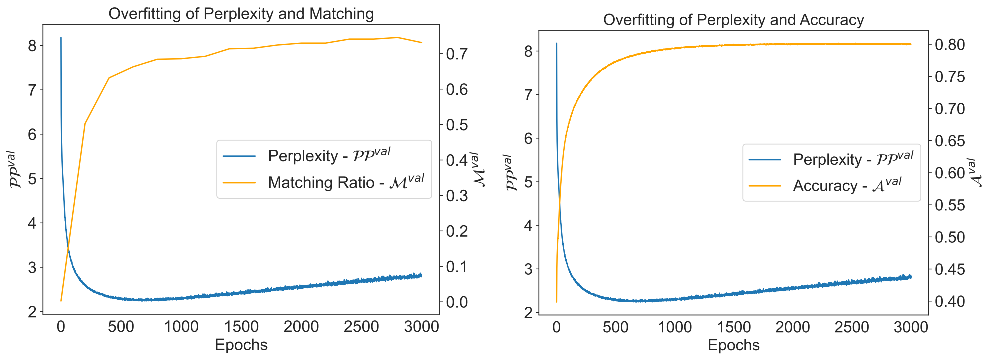

Fig. C.1. Evolution of the perplexity and the fraction of correctly matched pairs (a) on the validation set and (b) the accuracy during training for PF013171-PF14226 for the large Transformer.

B.5 Training Time

In this section, we present a table with the training time of our models using a single Nvidia V100 GPU. The first column refers to the family pair, the second to the training time of the shallow Transformer, the third one to the large Transformer with entropic regularization, and the last one to the large Transformer without entropic regularization.

Pair	Runtime Shallow	Runtime Renyi	Runtime Large
PF00289_PF02786	0 days 10:13:10	3 days 17:10:52	0 days 15:10:43
PF02785_PF02786	0 days 10:20:21	2 days 10:45:01	0 days 11:24:58
PF02785_PF00289	0 days 09:50:34	2 days 02:05:06	0 days 15:45:50
PF00004_PF07724	0 days 19:54:55	2 days 15:57:20	0 days 23:05:37
PF00006_PF02874	0 days 12:00:52	2 days 07:07:35	0 days 12:28:15
PF00207_PF07677	0 days 01:44:11	0 days 06:14:45	0 days 01:09:31
PF00207_PF01835	0 days 03:30:15	0 days 11:15:13	0 days 02:17:15
PF08264_PF00133	0 days 18:21:48	6 days 05:37:06	0 days 19:24:28
PF00501_PF13193	1 days 16:47:12	12 days 12:27:26	1 days 07:23:54
PF01591_PF00300	0 days 03:20:45	0 days 19:01:09	0 days 02:23:11
PF08240_PF00107	1 days 13:11:32	6 days 20:56:44	1 days 18:54:09
PF02770_PF02771	1 days 02:09:05	6 days 01:37:54	1 days 07:02:04
PF00441_PF02771	1 days 01:16:55	10 days 18:35:05	1 days 08:18:41
PF00441_PF02770	1 days 02:20:40	6 days 01:56:31	1 days 11:05:05
PF01842_PF13840	0 days 02:38:04	0 days 10:54:23	0 days 02:37:06
PF08545_PF08541	0 days 07:48:42	1 days 04:28:05	0 days 05:54:03
PF00005_PF08402	0 days 11:30:09	1 days 19:43:05	0 days 12:58:03
PF00005_PF08352	0 days 13:42:05	2 days 13:55:30	0 days 16:48:21
PF00664_PF00005	2 days 01:30:23	10 days 19:21:16	2 days 02:02:05
PF03171_PF14226	0 days 22:10:30	4 days 04:10:56	0 days 21:30:18
PF12780_PF12781	0 days 07:10:21	1 days 00:33:05	0 days 03:09:31
PF12775_PF12780	0 days 08:13:18	2 days 05:13:49	0 days 03:43:15
PF07724_PF10431	0 days 18:03:28	3 days 14:18:21	1 days 08:33:09
PF00690_PF00702	0 days 09:55:26	2 days 16:45:33	0 days 10:26:16
PF00690_PF00122	0 days 12:05:13	3 days 04:58:34	0 days 16:48:12
PF00689_PF00122	0 days 10:37:22	5 days 08:58:05	0 days 17:20:30
PF00004_PF10431	0 days 13:00:08	2 days 19:41:44	0 days 18:51:31
HKRR	0 days 9:03:08	2 days 15:41:14	0 days 15:38:34

Appendix C Regularization

C.1 Dropout and Weight-Decay Benchmark

In this section, we show learning curves related to overfitting behavior and regularization.

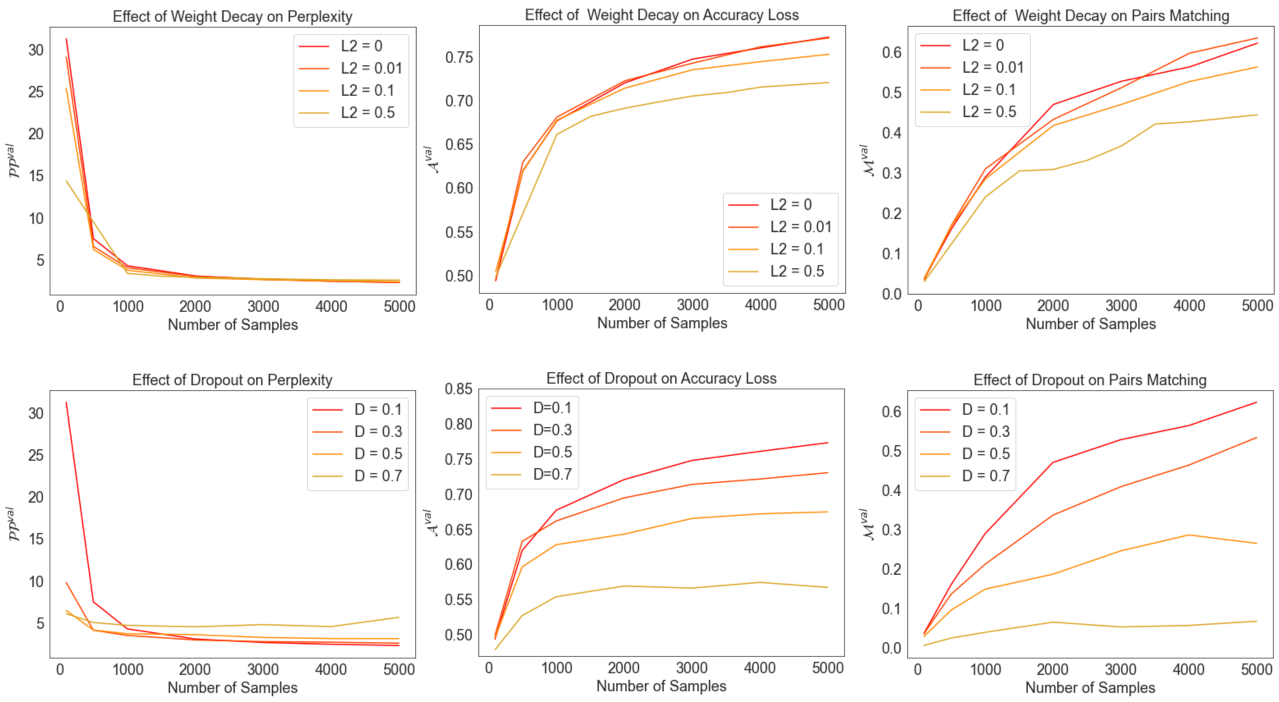

Fig. C.2. Performance of the Transformer on PF013171-PF14226 for a varying number of training examples: Left plots show the perplexity $\mathcal{PP}^{val}$, center plots show the accuracy $\mathcal{A}^{val}$, right plots show the fraction of correctly matched pairs $\mathcal{M}^{val}$. All values are for the validation set.

C.2 Entropic Formulation of The Regularization

There is a relation between the entropic regularization and the Rényi entropy: For a number of sampled sequences S sufficiently large we can rewrite the regularization term

$$\begin{aligned} R_{ent}(A_i, B_i) &= \log P(A_i|B_i) - \log S - \log\left(\frac{1}{S}\sum_{k=1}^{S} P(A_{i,k}|B_i) + \frac{1}{S}P(A_i|B_i)\right) \\ &\approx \log P(A_i|B_i) - \log S - \log\left(\mathbb{E}_{A\sim P(A|B_i)}[P(A|B_i)]\right) \\ &\approx \log P(A_i|B_i) - \log S - \log\left(\sum_A p^2(A|B_i)\right), \end{aligned} \tag{16}$$

where the last sum is over all possible sequences A. The first term in the last line is equivalent to the standard loss and can be absorbed there. The second term is a constant and will not influence the gradient. The last term is the logarithm of the Rényi entropy of order 2, also called the *collision entropy*, of the distribution over target sequences conditioned on B_i.

C.2.1 Entropic Regularization Performance

This section presents the results of the large Transformer trained with $\alpha = 0.7$, and $S = 5$ in comparison with arDCA and the shallow Transformer in Fig. C.3, Fig. C.4 and Fig. C.5. The comparison with the large Transformer without regularization or with weight decay is presented in the following section, see Appendix Sec. C.3.

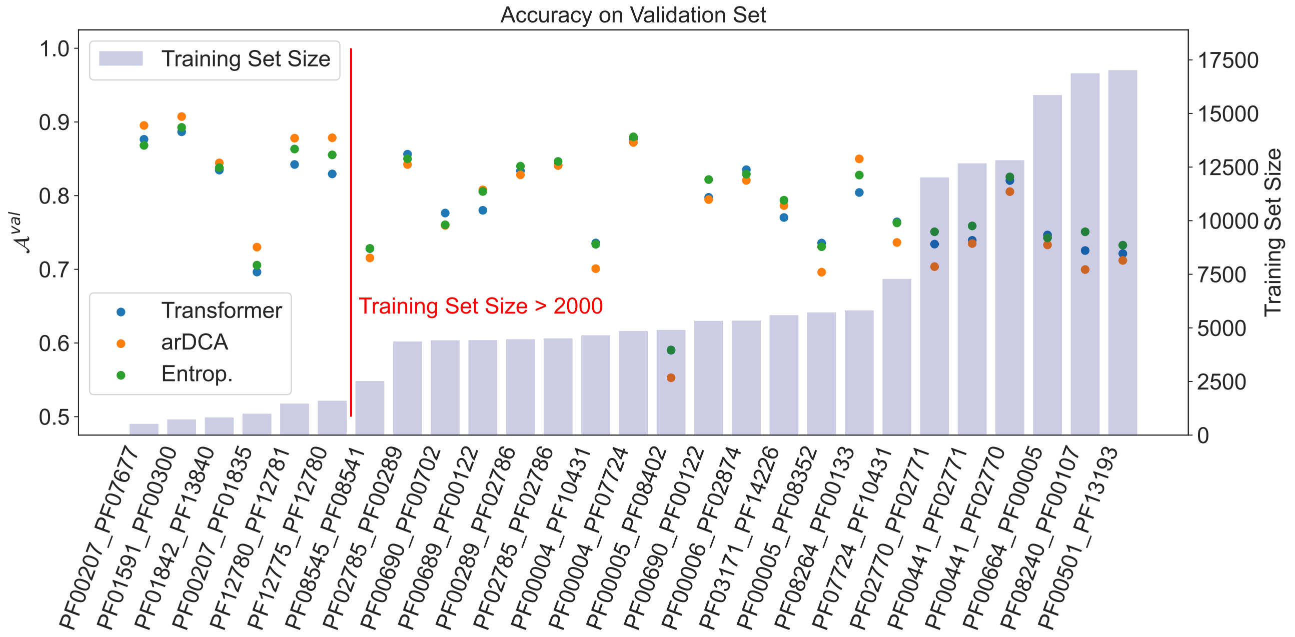

Fig. C.3. Accuracy $\mathcal{A}^{val}$ on the validation set for the shallow Transformer, arDCA, and the large Transformer with entropic regularization. The families are ordered by training set size. -For datasets below 2000 examples, arDCA is always above Transformer with an average difference of 0.02 in terms of accuracy -For datasets above 2000 examples, Transformer is below arDCA in 90.4% of cases with an average difference of 0.02 in terms of accuracy

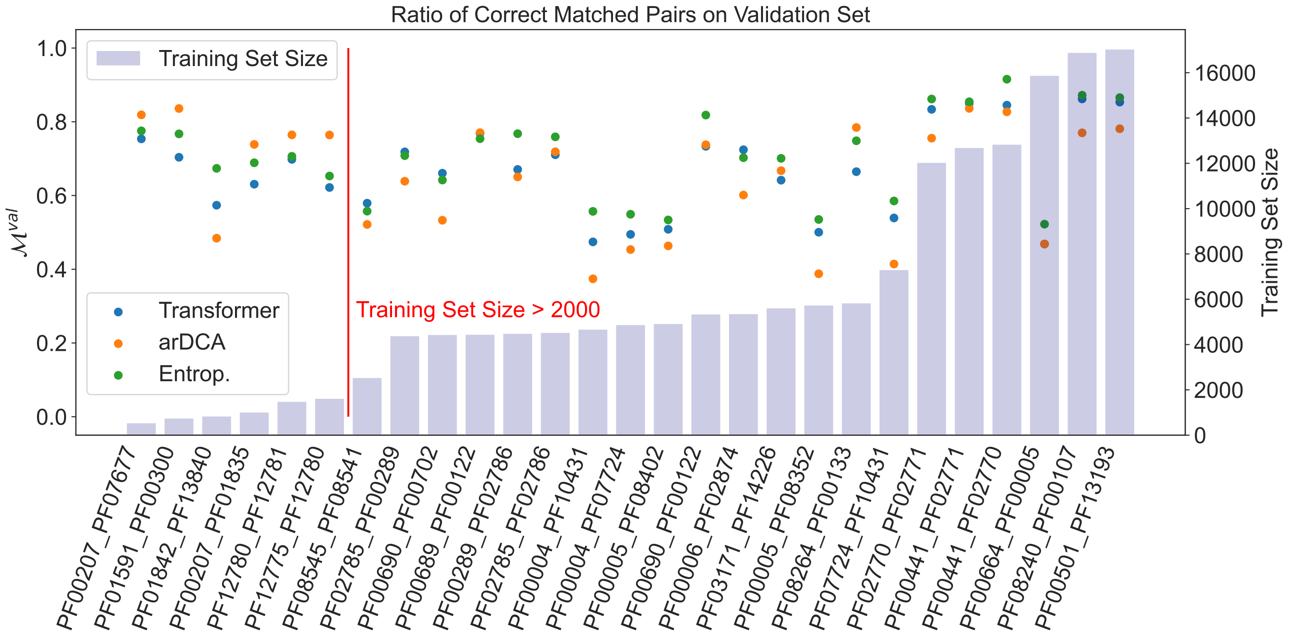

Fig. C.4. True positive rate for matching on the validation set for the shallow Transformer, arDCA, and the large Transformer with entropic regularization. The families are ordered by training set size. -For datasets below 2000 examples, arDCA is above Transformer in 83% of cases with an average difference of 0.02 in terms of matching fraction -For datasets above 2000 examples, Transformer is below arDCA in 90.4% of cases with an average difference of 0.08 in terms of matching fraction

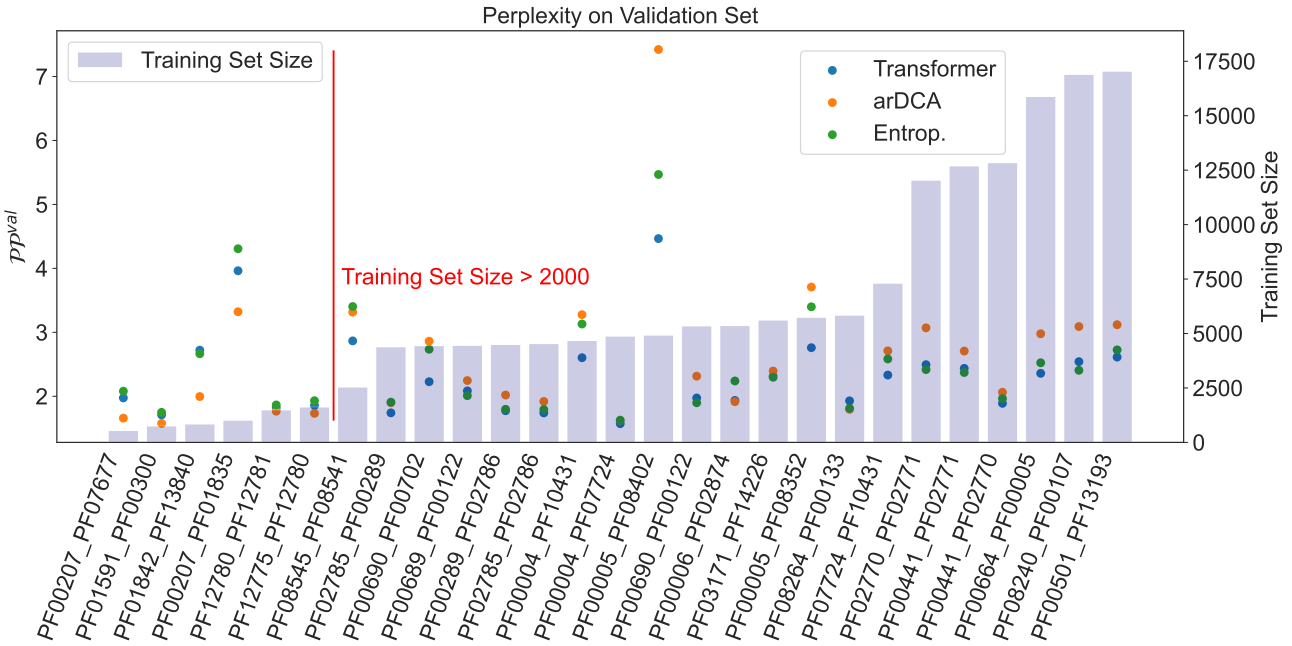

Fig. C.5. Perplexity $\mathcal{PP}^{val}$ for the shallow Transformer, the large Transformer with entropic regularization and arDCA on the validation set. The families are ordered by training set size. -For datasets below 2000 examples, arDCA is always below Transformer with an average difference of 0.42 in terms of perplexity -For datasets above 2000 examples, Transformer is below arDCA in 76% of cases with an average difference of 0.28 in terms of perplexity

C.3 Entropic Regularization Compared with Weight Decay

In this section, we compare the entropic regularization and weight decay on different metrics, averaged over all 27 families.

Entropic versus Weight Decay In this section, we compare the performance of the large Transformer on different metrics for all families individually. The resulting plots were split into different figures in order to make them fit on the pages.

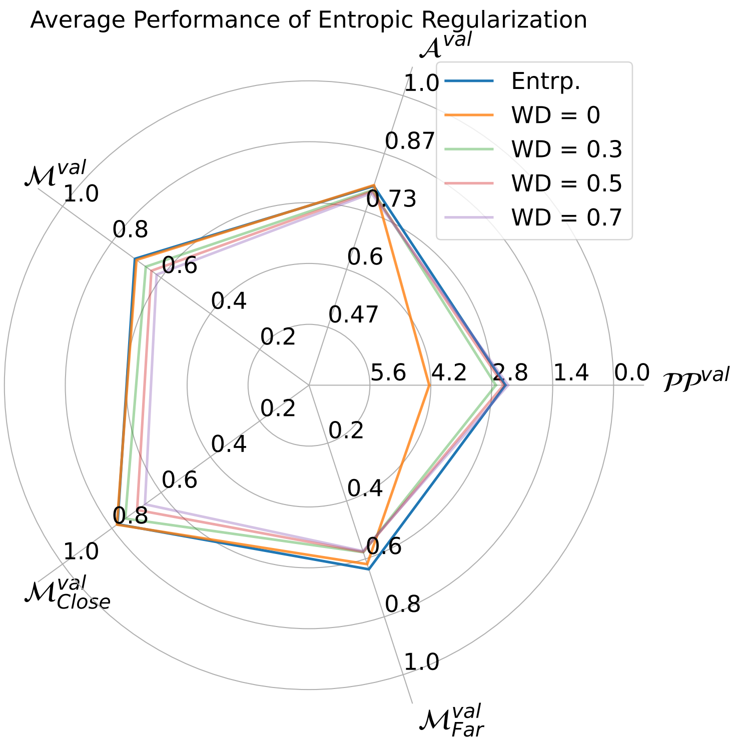

Fig. C.6. Radar plot comparing different regularization schemes, entropic (Entrp.), and weight decay (WD) with different strengths for the large Transformer. The radial direction of the perplexity $\mathcal{PP}$ is reversed in order to have the same direction for increasing performance as for the other metrics. The plot was done by averaging the metrics of all families.

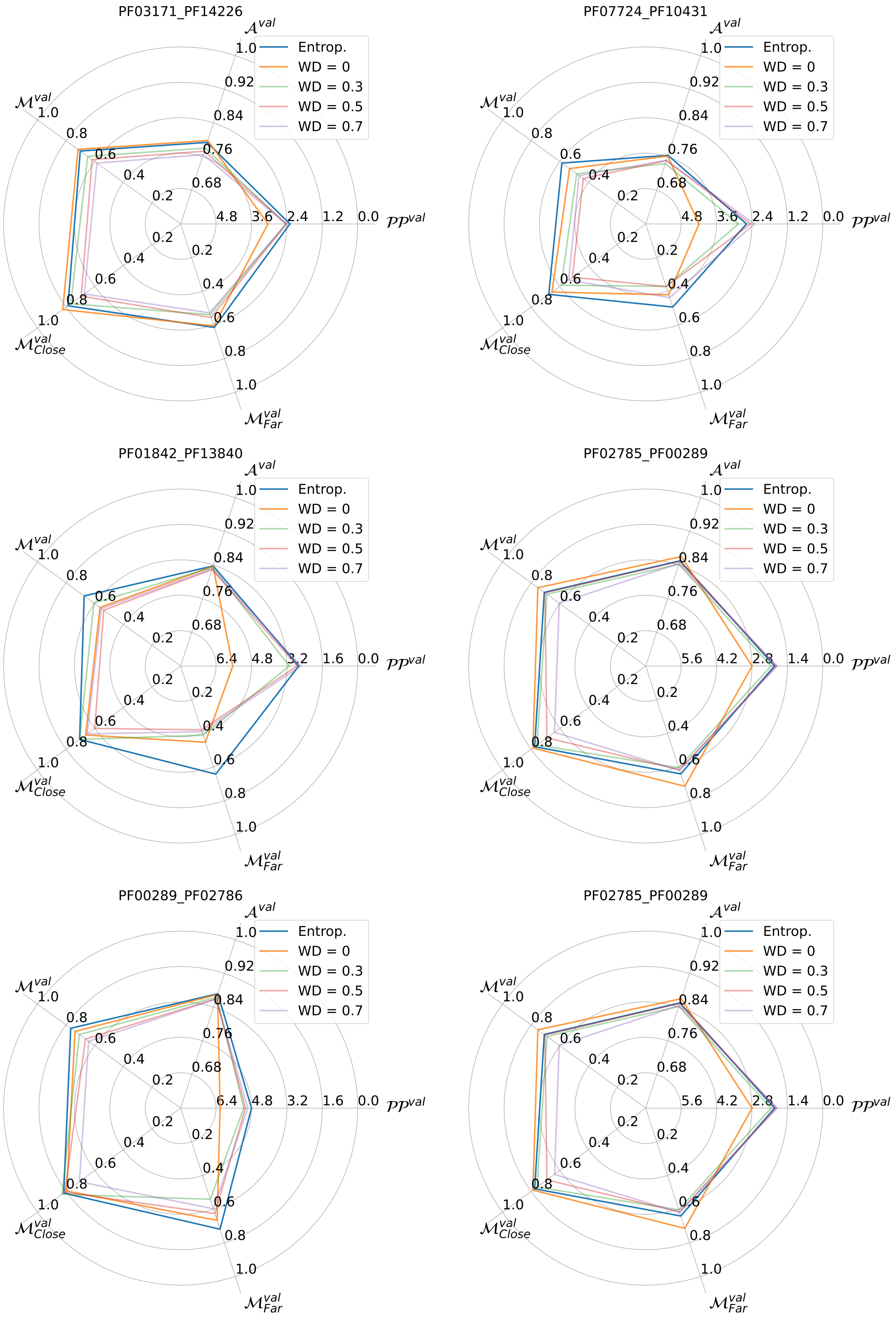

Fig. C.7. Comparing the performance of the large Transformer with entropic regularization and weight decay (WD). The radial direction of the perplexity $\mathcal{PP}$ is reversed in order to have the same direction for increasing performance as for the other metrics.

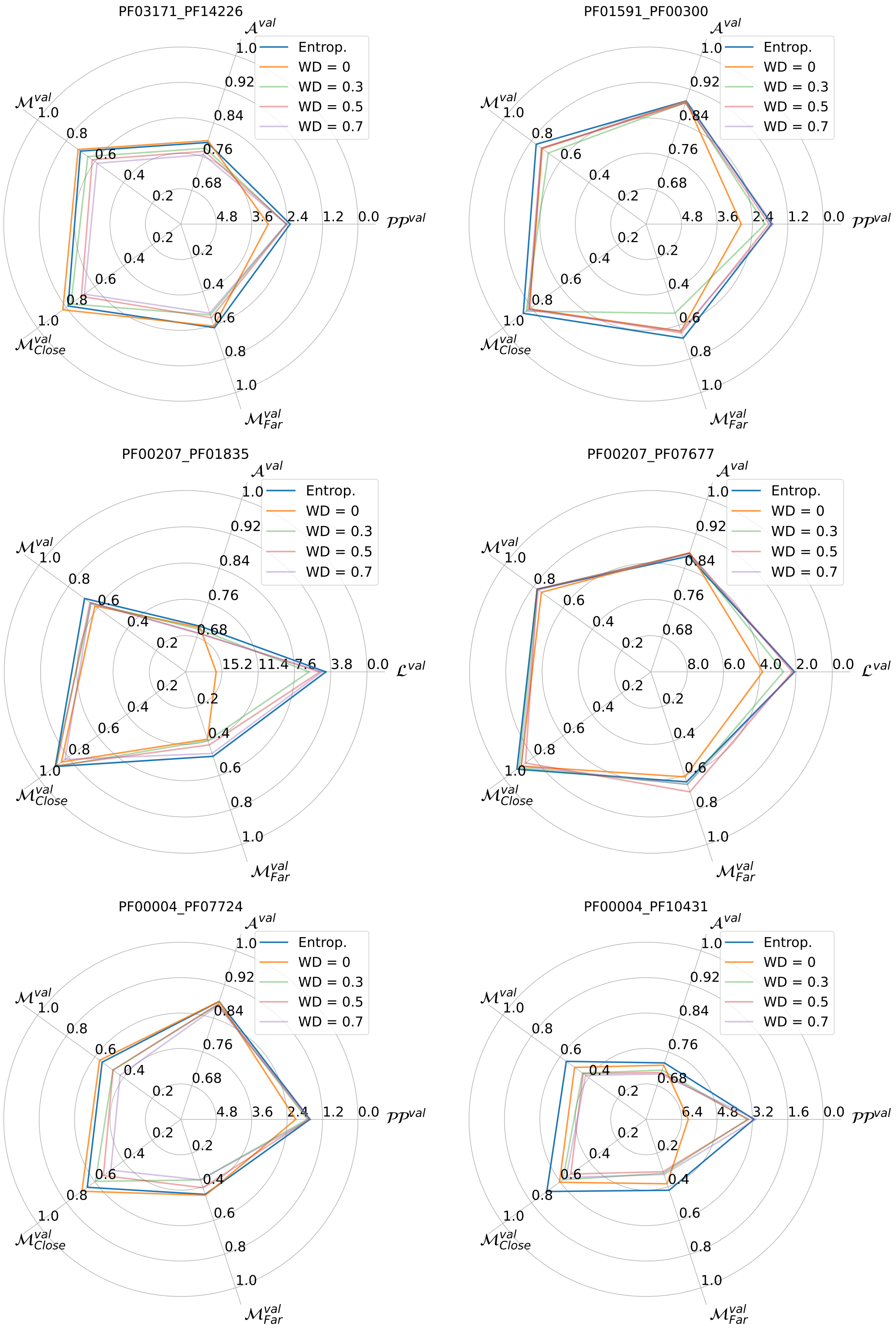

Fig. C.8. Comparing the performance of the large Transformer with entropic regularization and weight decay (WD). The radial direction of the perplexity $\mathcal{PP}$ is reversed in order to have the same direction for increasing performance as for the other metrics.

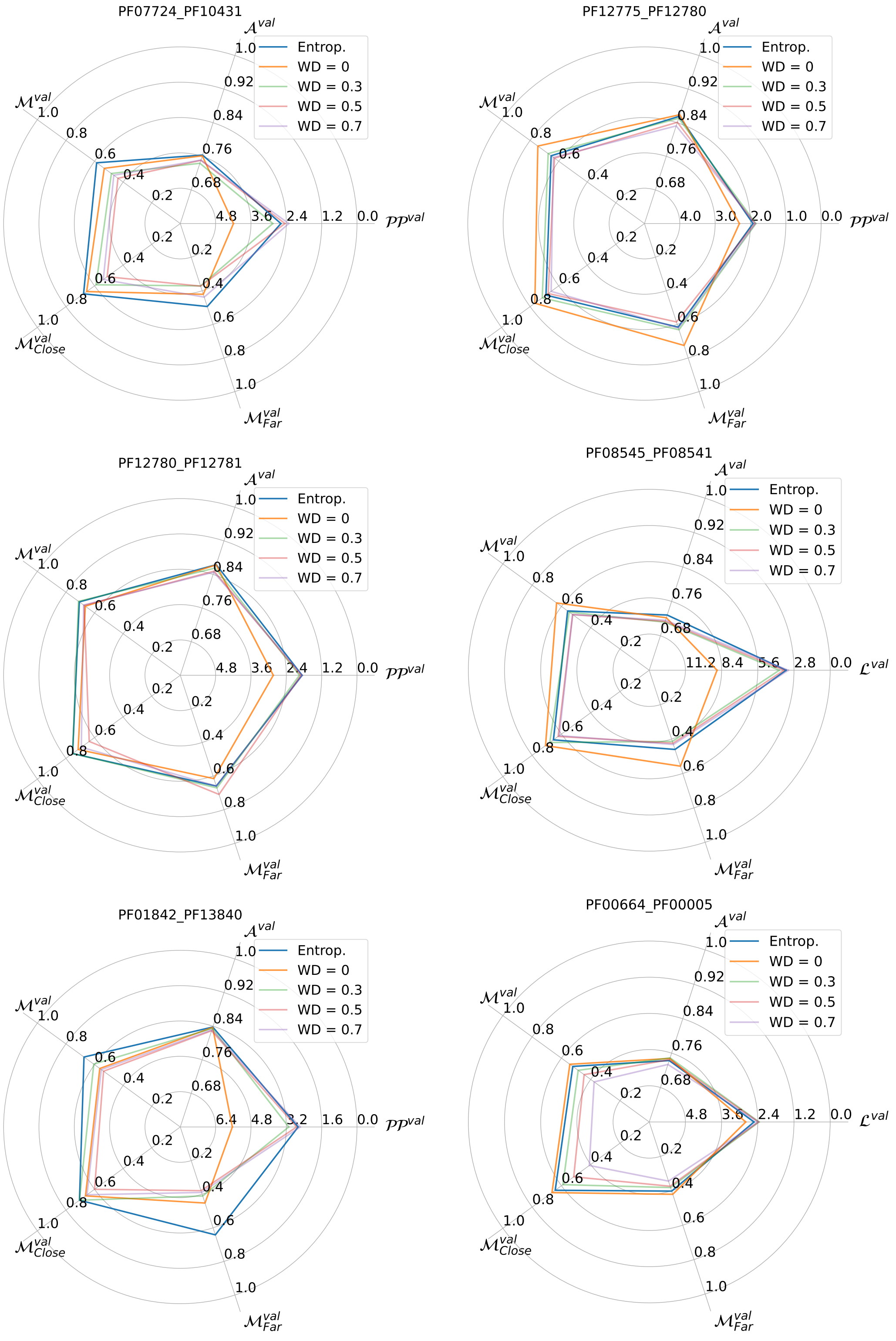

Fig. C.9. Comparing the performance of the large Transformer with entropic regularization and weight decay (WD). The radial direction of the perplexity $\mathcal{PP}$ is reversed in order to have the same direction for increasing performance as for the other metrics.

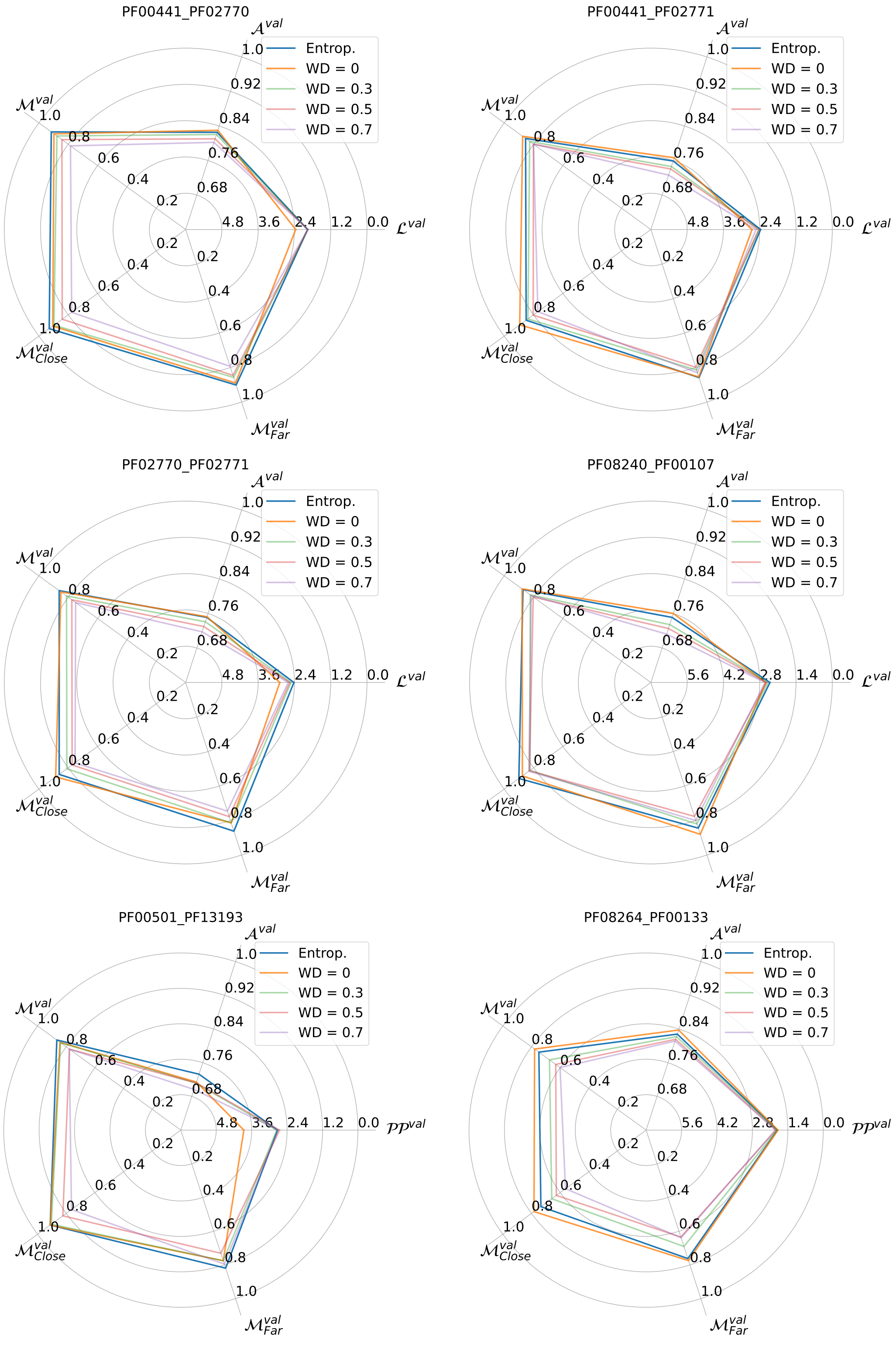

Fig. C.10. Comparing the performance of the large Transformer with entropic regularization and weight decay (WD). The radial direction of the perplexity $\mathcal{PP}$ is reversed in order to have the same direction for increasing performance as for the other metrics.

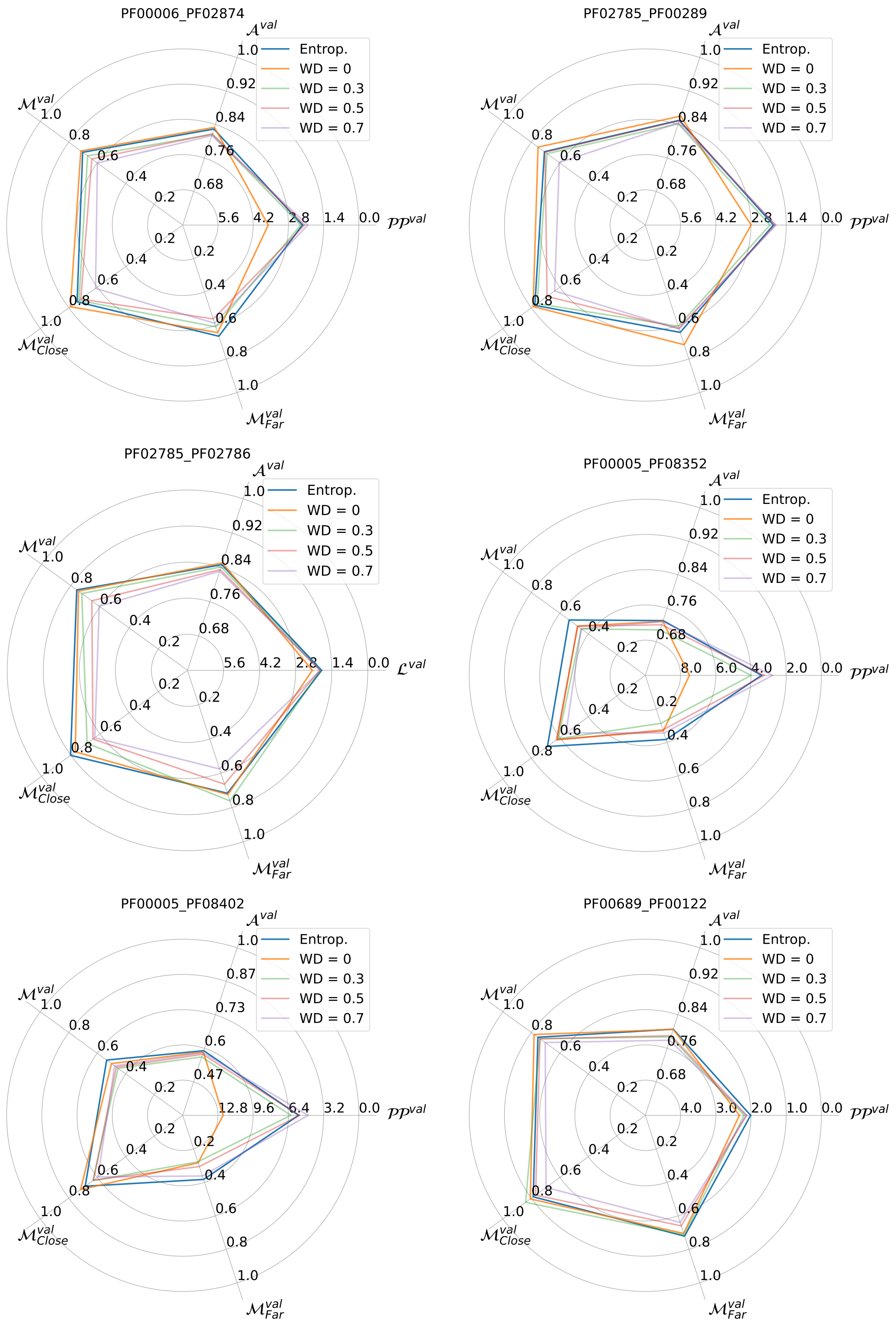

Fig. C.11. Comparing the performance of the large Transformer with entropic regularization and weight decay (WD). The radial direction of the perplexity $\mathcal{PP}$ is reversed in order to have the same direction for increasing performance as for the other metrics.

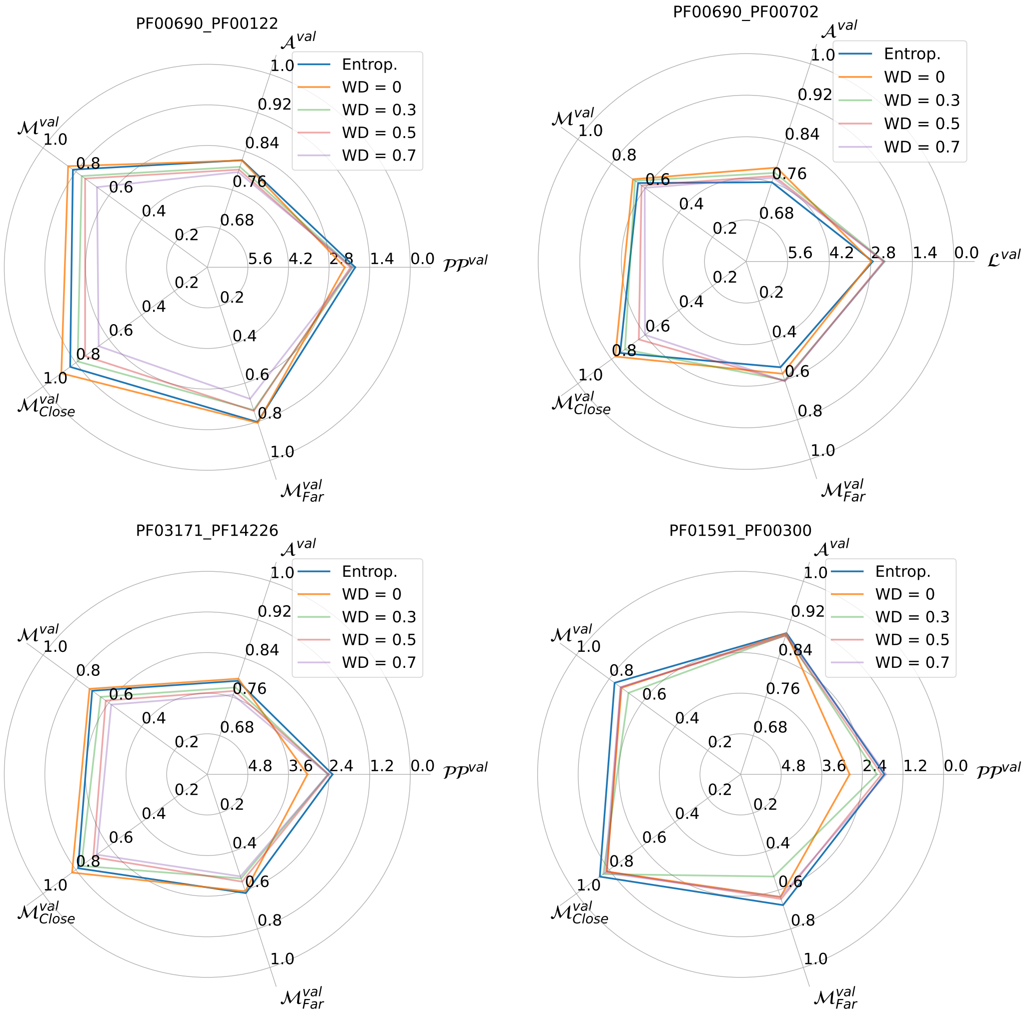

Fig. C.12. Comparing the performance of the large Transformer with entropic regularization and weight decay (WD). The radial direction of the perplexity $\mathcal{PP}$ is reversed in order to have the same direction for increasing performance as for the other metrics.

Appendix D Additional Structural Results

D.1 Structural comparison using AlphaFold

For all our computations we used the implementation of CollabFold with template search, 5 models, and 3 recycle. (Mirdita *et al.*, 2022).

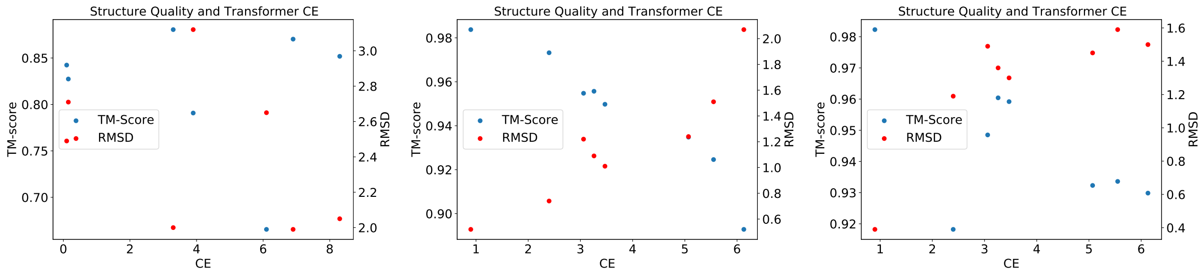

Fig. D.1. TM-Scores and RMSD values when comparing the Alphafold predicted structures of true sequences with Alphafold predicted structures of sequences where single domains have been replaced with homologous natural sequences. (Left is based on G0S4G4, which contains domains PF00004 and PF07724, which are in contact in PDB 5D4W. Homologous sequences are sampled from the validation set and inserted into the G0S4G4 sequence. Center is based on Q8ZN46, which contains domains PF00207 and PF01835, which are in contact in PDB 4U4J. Homologous sequences are sampled from the validation set and inserted into the Q8ZN46 sequence. Right is based on Q13SV4, which contains domains PF08545 and PF08541, which are in contact in PDB 4EFI. Homologous sequences are sampled from the validation set and inserted into the Q13SV4 sequence. In all of these proteins, we measure the change in structural scores and in cross-entropy in the shallow Transformer model (abscissa).

D.2 Structural Information using DCA

Direct Coupling Analysis is a group of unsupervised methods for modeling aligned protein sequences, see Ref. (Cocco *et al.*, 2018). Apart from other applications, it can be used for predicting structural contacts from MSAs.

plmDCA plmDCA is a specific method of DCA based on a pseudolikelihood approximation for training the Potts Model. In this paper we used the asymmetric version of the method from https://github.com/pagnani/ArDCA.jl with default hyperparameters. The sequences sampled from the Transformer were realigned using HMMer (Finn *et al.*, 2011).

Results per Pairs In this section, we show the contact prediction results obtained with plmDCA for the 27 families.

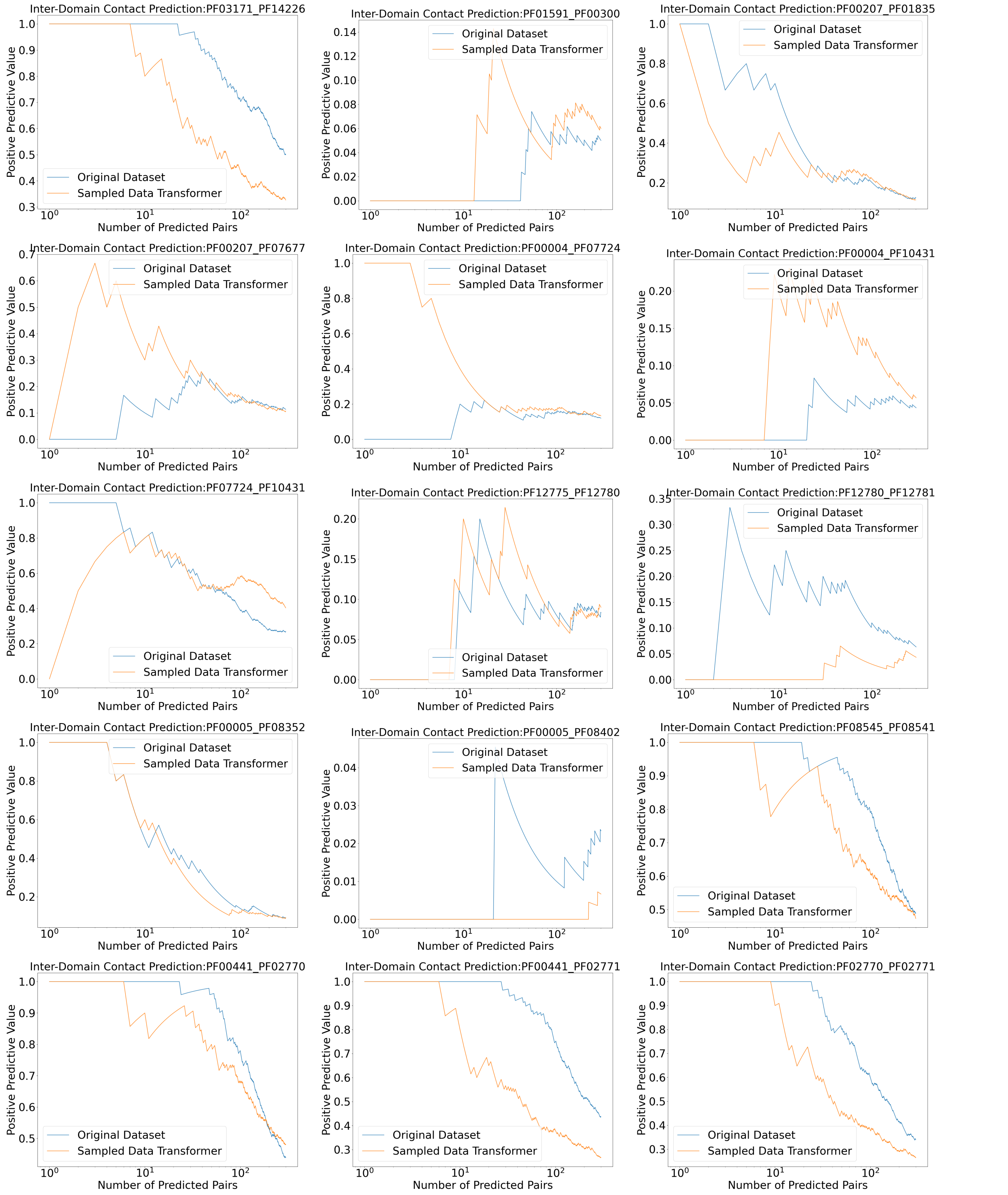

Fig. D.2. Contact Prediction using plmDCA for the original training set, and a sampled dataset from the shallow Transformer. The curves represent the Positive Predictive Value (fraction of true positives) with respect to the number of predicted contacts. To make it fit the page format, we split the results on the different families into two figures: this one and the following.

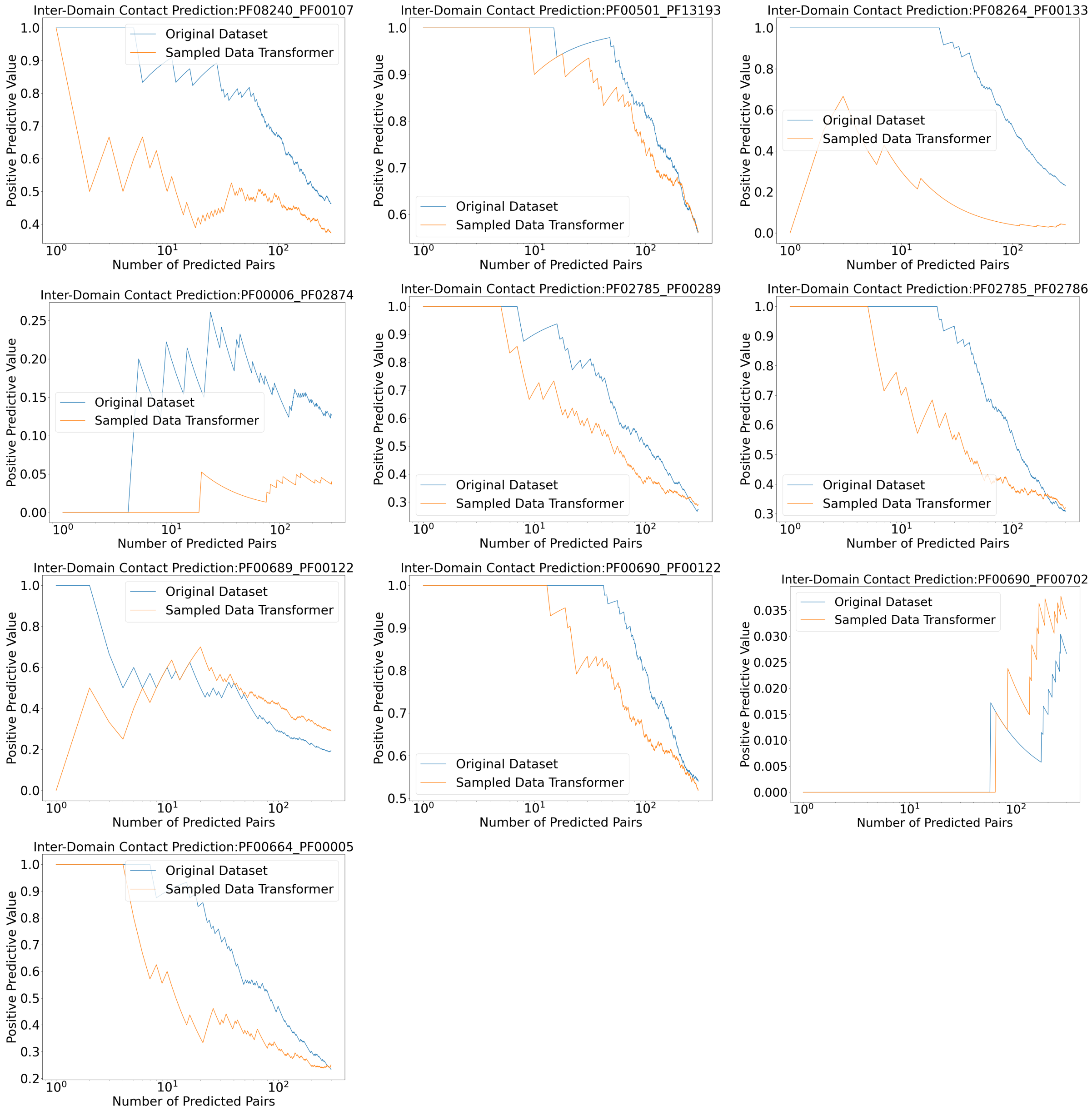

Fig. D.3. Contact Prediction using plmDCA for the original training set, and a sampled dataset from the shallow Transformer. The curves represent the Positive Predictive Value (fraction of true positives) with respect to the number of predicted contacts.

Appendix E Additional Results on Generalization

E.1 Matching Performance for Different Distances from Training Set

Here we plot the fraction of correctly matched pairs in the validation set, separated into below-median and above-median distances from the training set.

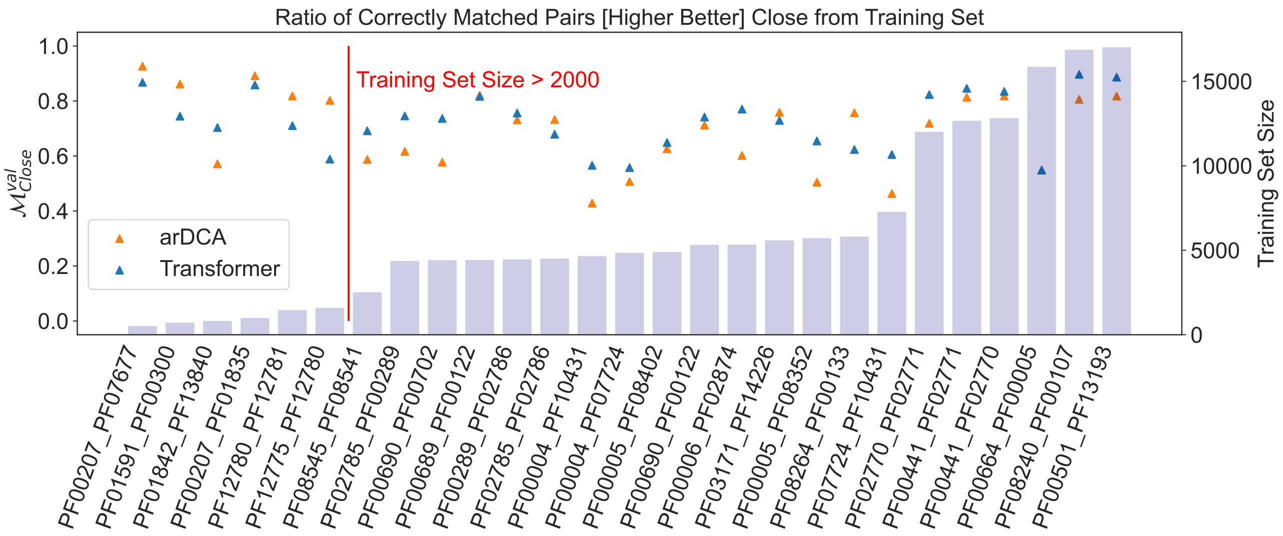

Fig. E.1. Fraction of correctly matched pairs in the validation set for the shallow Transformer and arDCA. The families are ordered by training set size. Shown are results for the 50% of sequences closest to the training set.

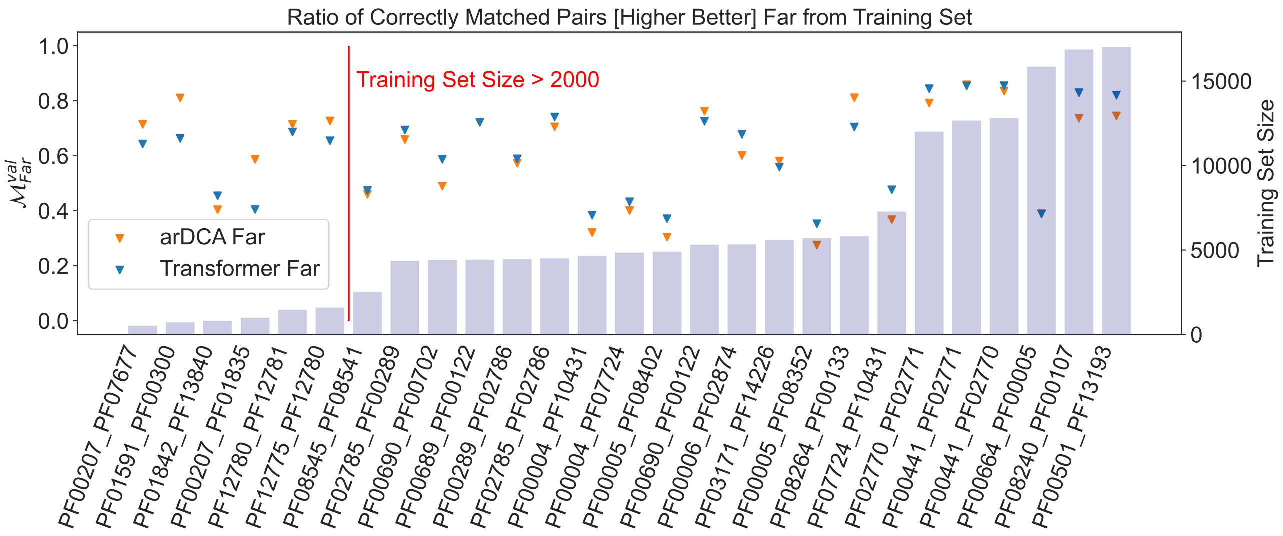

Fig. E.2. Fraction of correctly matched pairs in the validation set for the shallow Transformer and arDCA. The families are ordered by training set size. Shown are results for the 50% of sequences farthest to the training set.

E.2 Accuracy and Perplexity with Distance from Training Set

In this section, we present the results for each pair of the analysis of Sec. 4.5.

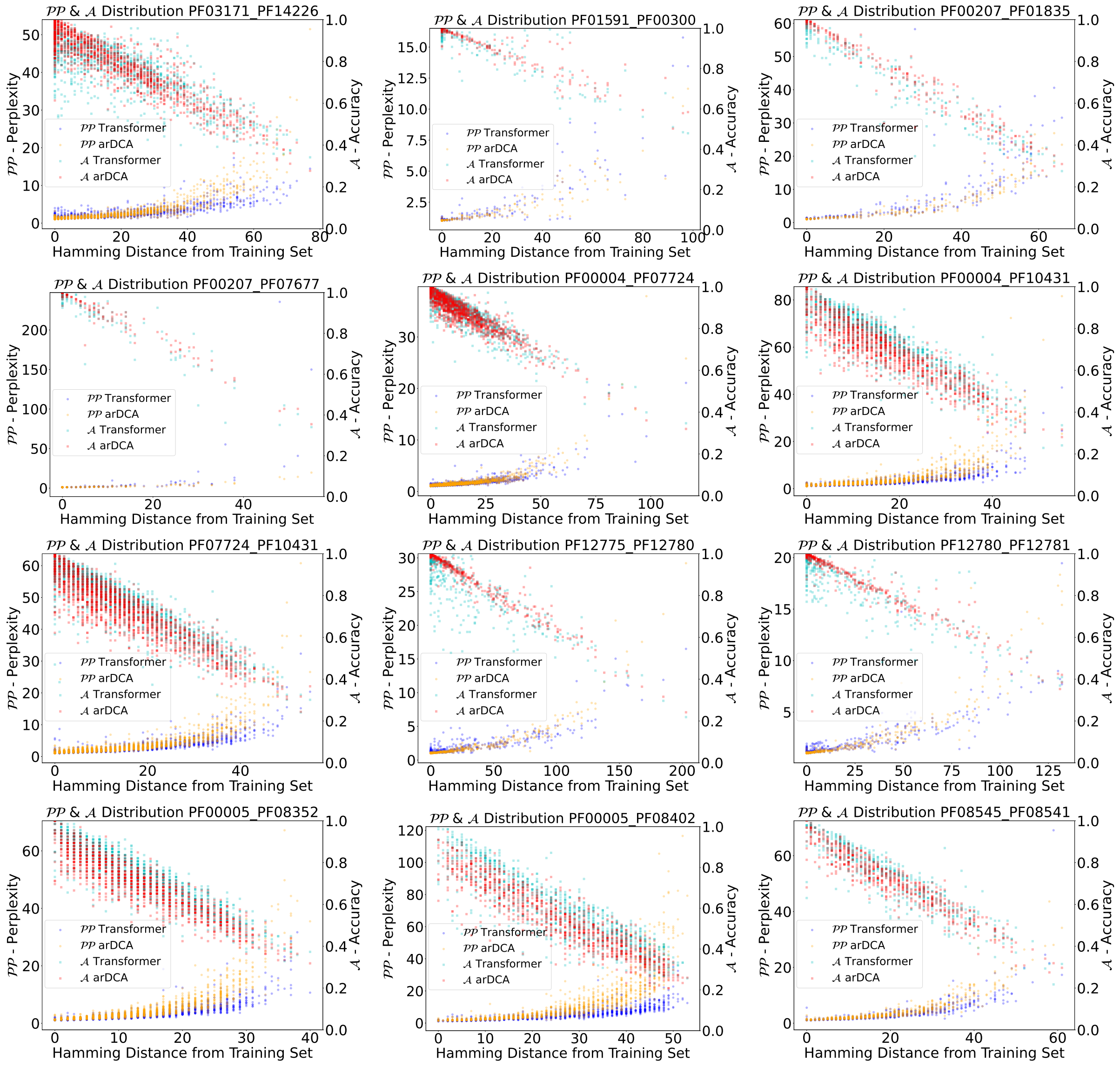

Fig. E.3. Distribution of the perplexity $\mathcal{PP}$ and the accuracy $\mathcal{A}$ of every sequence pair in the validation set with respect to their distance from the training set for the shallow Transformer and arDCA. To fit the page format, we split the results on the different families into two figures: this one and the following

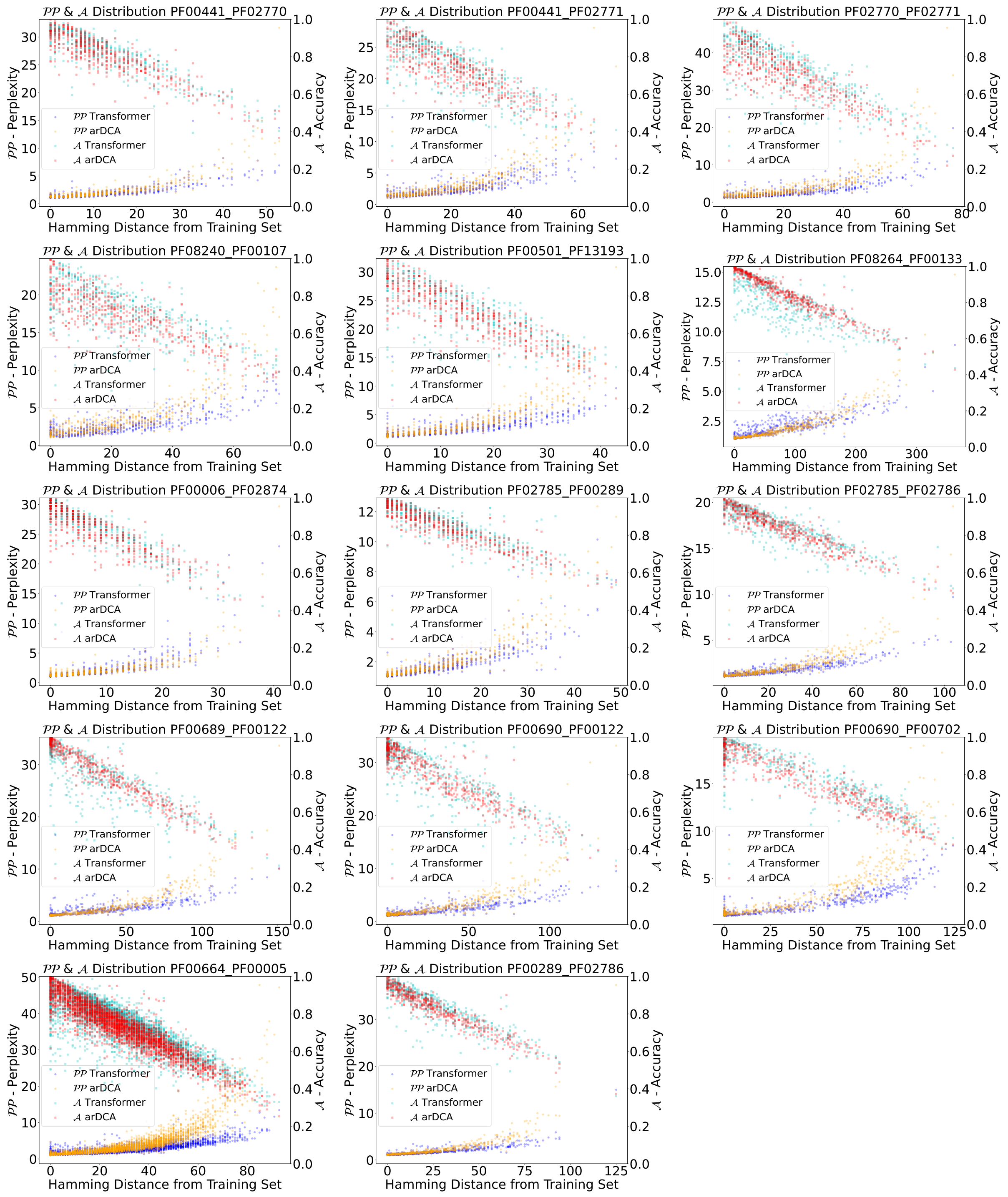

Fig. E.4. Distribution of the perplexity $\mathcal{PP}$ and the accuracy $\mathcal{A}$ of every sequence pair in the validation set with respect to their distance from the training set for the shallow Transformer and arDCA.

Appendix F Additional Matching evaluation

For each protein family, we measure the fraction of correctly matched pairs when restricting the problem to the first n sequence pairs.

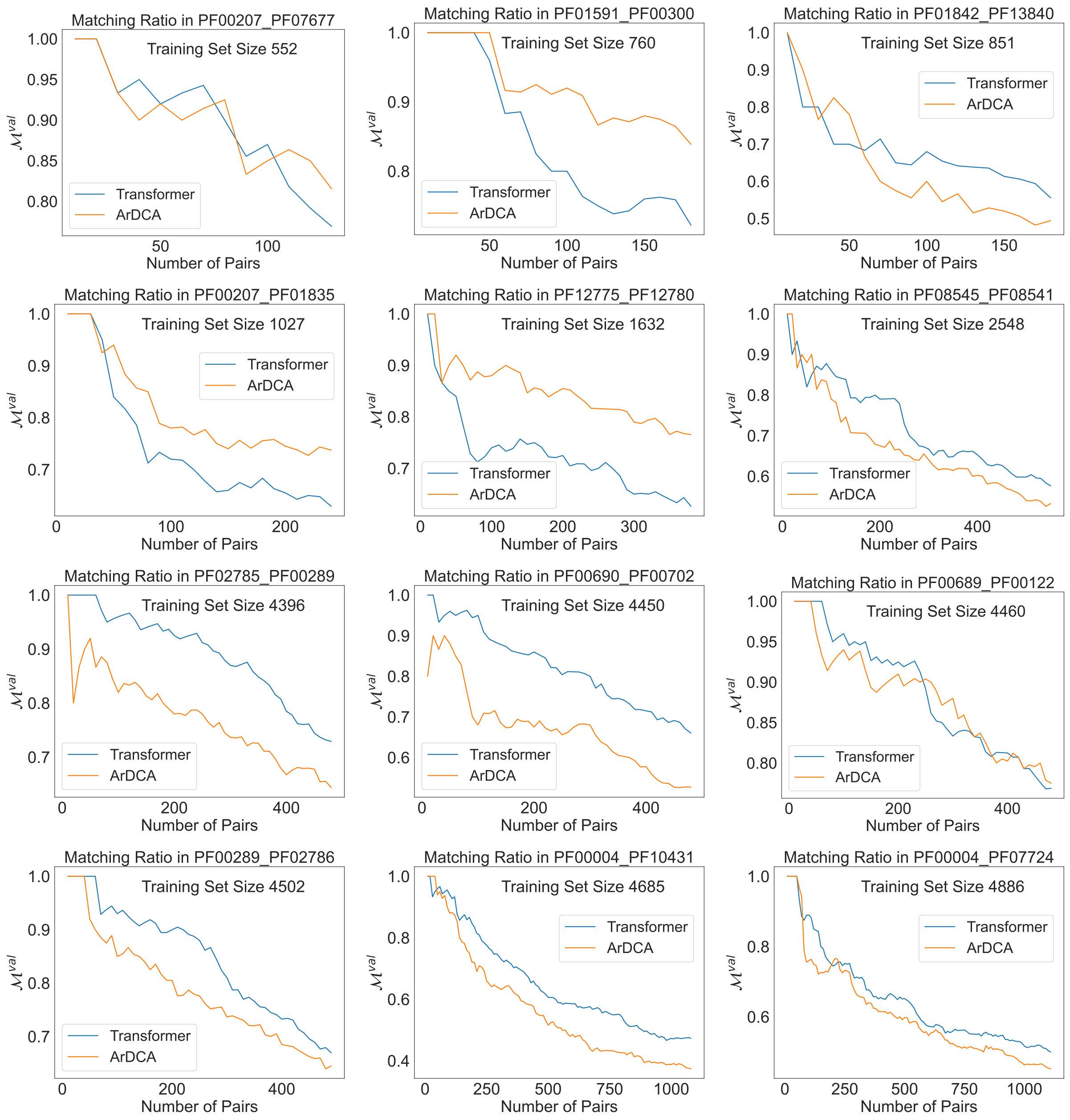

Fig. F.1. Fraction of correctly matched pairs $\mathcal{M}^{val}$ for increasing number of pairs for different families. Shown are the results for the shallow Transformer (blue) and arDCA (orange). Only a subset of the families is shown in order to save computational resources. The families are ordered according to the training set size. In order to fit the page format, we split the results into this and the next figure.

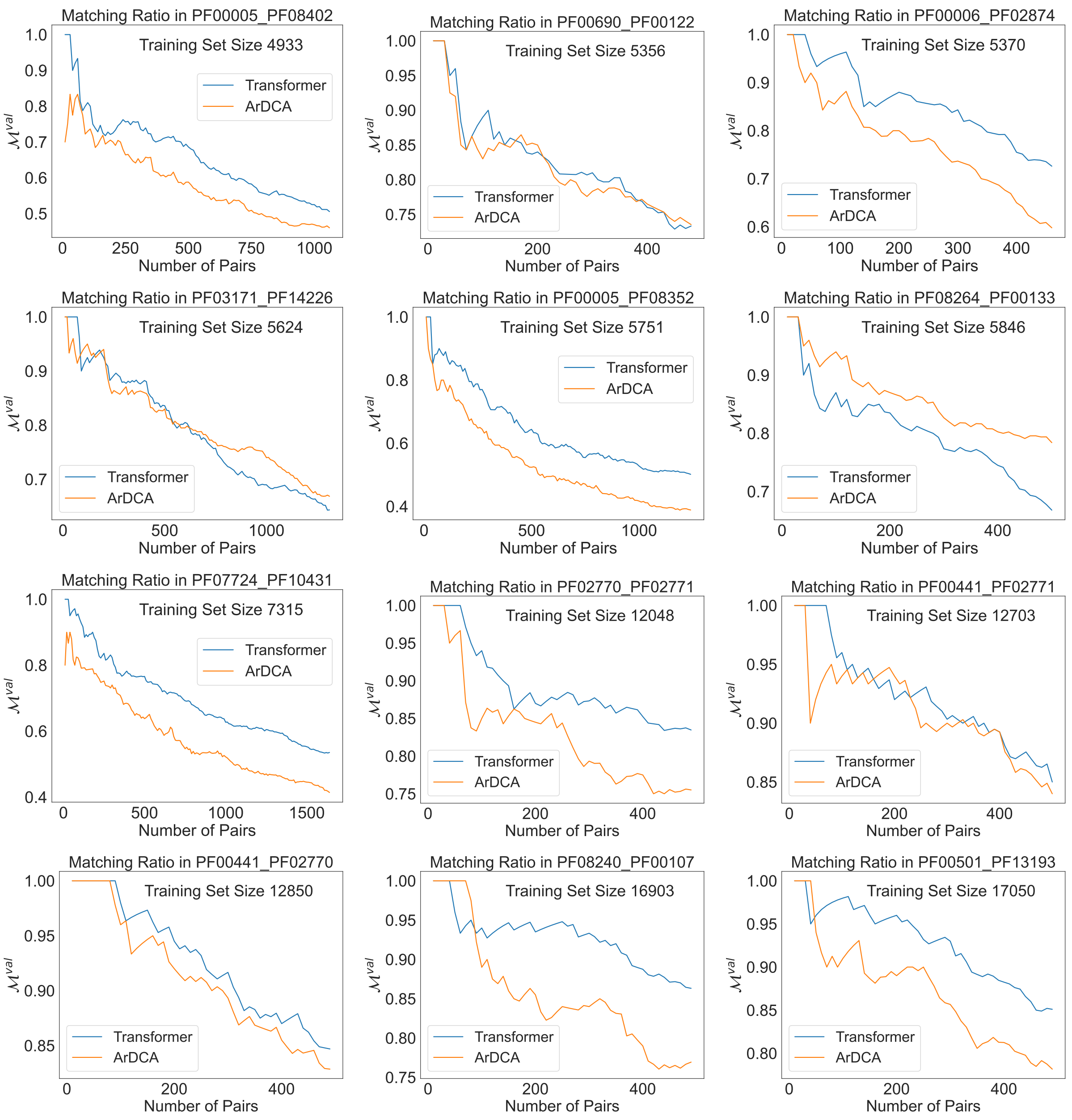

Fig. F.2. Fraction of correctly matched pairs $\mathcal{M}^{val}$ for increasing number of pairs for different families. Shown are the results for the shallow Transformer (blue) and arDCA (orange). Only a subset of the families is shown in order to save computational resources. The families are ordered according to the training set size. In order to fit the page format, we split the results into this and the previous figure.

F.1 D

F.1.1 D.3 Sequence Logo and Loss

In Fig. F.3 we show the perplexity per position for family pair PF013171-PF14226, together with the sequence logo. It is evident that biologically conserved positions correspond to lower perplexities, which is to be expected.

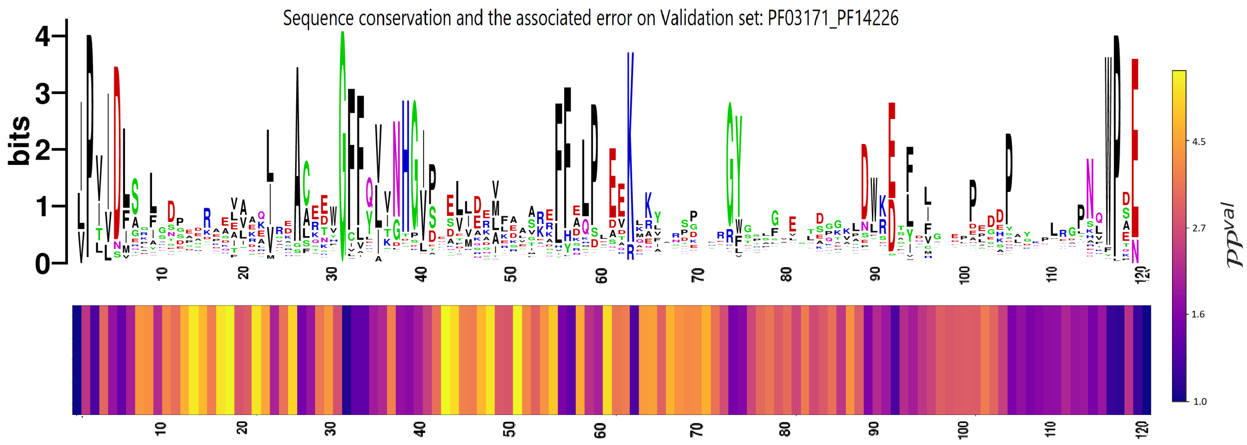

Fig. F.3. Top: Sequence logo of PF013171-PF14226 paired MSA. Bottom: Distribution of the perplexity with respect to the positions. The errors are concentrated on the most variable position, highlighting that the Transformer has understood the basic site-wise structure of the distribution.

Supplementary References
